# Genetic analyses and functional validation of ruminant SLAMs reveal potential hosts for PPRV

**DOI:** 10.1186/s13567-025-01489-w

**Published:** 2025-03-18

**Authors:** Xi Wei, Kejia Lu, Zhengwu Chang, Hanwei Guo, Qinfeng Li, Binxuan Yuan, Chen Liu, Zengqi Yang, Haijin Liu

**Affiliations:** 1 College of Veterinary Medicine, Northwest A&F University, Yangling, 712100 Shaanxi China; 2 Engineering Research Center of Efficient New Vaccines for Animals, Ministry of Education, Yangling, China; 3 Key Laboratory of Ruminant Disease Prevention and Control (West), Ministry of Agriculture and Rural Affairs, Yangling, China; 4 Engineering Research Center of Efficient New Vaccines for Animals, Universities of Shaanxi Province, Yangling, China

**Keywords:** PPRV, SLAM, ruminant animals, host range

## Abstract

Peste des petits ruminants (PPR), caused by the peste des petits ruminants virus (PPRV), is a highly contagious disease affecting ruminants. While goats and sheep are well-known hosts, PPRV has also spread to wild ruminants, and it remains unclear which ruminant species can be infected. SLAM (Signaling lymphocytic activation molecule) acts as the primary receptor for PPRV, playing a crucial role in the viral infection process. Identifying which ruminant SLAMs can mediate PPRV infection is essential for understanding the potential hosts of PPRV, which is vital for effective eradication efforts. In this study, we first extracted 77 ruminant species’ SLAM sequences from ruminant genome database. Based on these sequences, we predicted the structures of ruminant SLAMs. The analysis revealed that SLAM conformation is similar across ruminant species, and the potential PPRV H protein binding domain residues were conserved among SLAMs of these 77 species. Phylogenetic analysis of SLAM grouped ruminants into six families. We then selected representative SLAMs from each ruminant family to assess their role in PPRV infection. Our findings demonstrated that ruminant SLAMs efficiently mediated PPRV infection, with enhanced viral amplification observed in cells expressing SLAM from java mouse deer (*Tragulidae*) and goat (*Bovidae*), compared to cells expressing SLAM from white tailed deer (*Cervidae*) and giraffe (*Giraffidae*). These results underscore the need to consider a broader range of potential host populations beyond goat and sheep in efforts to prevent and eradicate PPRV.

**Supplementary Information:**

The online version contains supplementary material available at 10.1186/s13567-025-01489-w.

## Introduction

Peste des petits ruminants (PPR) is a highly contagious viral disease affecting small ruminants, caused by the peste des petits ruminants virus (PPRV). PPRV, a member of the genus *Morbillivirus* in the family *Paramyxoviridae*, is an enveloped RNA virus with a non-segmented, negative-sense, single-stranded genome [1]. In terms of genome length, PPRV is noted as the largest member within the *Morbillivirus* genus, with 15,948 base pairs [2]. The genome encodes six structural proteins: nucleocapsid protein (N), RNA-dependent RNA polymerase (L), RNA polymerase phosphoprotein cofactor (P), matrix protein (M), fusion protein (F), and hemagglutinin protein (H) [3]. PPRV primarily infects ruminant animals, notably sheep and goats, and is characterized by clinical symptoms including fever, pneumonia, diarrhea, and inflammation of the respiratory and digestive tracts [4]. The disease can result in morbidity and mortality rates approaching 100% [5]. Consequently, PPR has significant socio-economic impacts on the livestock sector, particularly in economically dependent and least developed countries. Following the successful global eradication of rinderpest in 2011, the Food and Agriculture Organization of the United Nations (FAO) and the World Organisation for Animal Health (WOAH) have prioritized PPR as the next target for global eradication efforts [6].

The ruminants are divided into six families: *Tragulidae*, *Moschidae*, *Cervidae*, *Antilocapridae*, *Giraffidae* and *Bovidae* [7]. Although PPR outbreaks are rarely reported in ruminants other than goats and sheep, PPRV has been documented to cause fatalities in wild bighorn sheep, saiga antelope, Asian water buffalo and camels, suggesting a potential spillover of PPRV to wild ruminants [8–11]. Consequently, to effectively eradicate PPRV, it is crucial to determine whether other ruminant species are susceptible to PPRV infection. The receptor plays a pivotal role in viral infection. PPRV binds to receptors on host cells via the H protein, which facilitates viral attachment to the cell surface [12]. This binding induces conformational changes in the H protein, thereby activating the fusion (F) protein and leading to the fusion of the viral and host cell membranes [13, 14].

The signaling lymphocyte activation molecule (SLAM), also known as CD150, and the cell adhesion molecule Nectin-4 (also known as poliovirus receptor-like protein 4, PVRL-4) are the primary cellular receptors required for the attachment of PPRV to host cells [14, 15]. Specifically, SLAM mediates infection in immune cells and facilitates virus spread, while Nectin-4 is responsible for mediating infection in epithelial cells [16]. SLAM expressing on the surface of lymphocytes, serves as the primary receptor for morbilliviruses, including measles virus (MV), rinderpest virus (RPV), canine distemper virus (CDV), and PPRV [17]. Sarkar et al. compared the amino acid sequences of SLAM from goats, sheep, cattle, and water buffalo, revealing high homology between goat SLAM and sheep SLAM (96.8%), followed by cattle (93.5%) and water buffalo (92.9%) [18]. Ohishi et al. identified eight key amino acid residues in the V domain of goat SLAM that influence host-virus specific binding, which are fully conserved in sheep, cattle, and water buffalo [19]. Reports indicate that PPRV infection has been observed in these species, but over the past few decades, the virus has expanded its host range to many non-natural hosts through mechanisms that remain unclear [20, 21]. This suggests that PPRV possesses the potential to adapt to various new hosts, which could impact the effectiveness of global eradication efforts for PPR.

Understanding whether SLAM from different ruminant species mediate PPRV infection is crucial for elucidating the progression of animal infections. In this study, we analyzed SLAM from 77 ruminant animals and found it to be highly conserved, particularly in regions potentially involved in interactions with the viral H protein. Furthermore, cells expressing SLAMs from each ruminant family effectively mediated PPRV infection and replication. These findings indicate that SLAM from various ruminant species can facilitate PPRV infection, suggesting that PPRV has the potential to infect additional host species beyond goats and sheep. Consequently, these potential host populations should be considered in PPRV prevention and eradication efforts.

## Materials and methods

### Cells and virus

Human renal HEK-293T cells were maintained in high-glucose Dulbecco’s Modified Eagle’s Medium (DMEM, Gibco) supplemented with 10% fetal bovine serum (FBS, Gibco) and incubated at 37 °C with 5% CO_2_. The Vero cell line stably expressing Dog-SLAM was constructed in our laboratory in a previous study and cultured in Minimum Essential Medium (MEM, Gibco) containing 10% FBS and incubated at 37 °C with 5% CO_2_. In prior work, we established a reverse genetics system for the PPRV vaccine strain (Nigeria75/1) based on the methodology described by Hu et al. [22]. By inserting the GFP gene between the P and M genes, we rescued the rPPRV-GFP (GFP-expressing) strain, which is stored at −80 °C.

### Phylogenetic analysis and sequence alignment

The coding sequence (CDS) region of the goat SLAM gene was downloaded from the National Center for Biotechnology Information (NCBI) database. Exon sequences of SLAM genes from 76 other ruminants were extracted from the ruminant genome database based on the position of goat SLAM on goat chromosome 3 [7, 23]. The nucleotide sequences of 77 SLAM orthologs with potential viral receptor activity were analyzed using MEGA-X (v10.05) software. Sequence alignment was performed with the MUSCLE algorithm. The alignment file was used to construct a phylogenetic tree using the neighbor-joining method with default parameters in MEGA-X. The phylogenetic tree was visualized using TVBOT software [24]. Amino acid sequences of SLAM across different species were compared and analyzed using EsPript 3.0 [25].

### Protein structure analysis

Using DMFold—Multimer to predict goat SLAM and PPRV H interactions, and protein structure of six SLAM homologue, use PyMOL (The PyMOL Molecular Graphics System, Version 1.7.4 Schrödinger, LLC) on protein structure comparison and annotations [26].

### Establishment of a stable 293T cell line expressing SLAM receptors

SLAM genes from java mouse deer (JMD), giraffe, Chinese forest musk deer (CFMD), white tailed deer (WTD), pronghorn (PH) and goat (MG669626.1) were synthesized. Codon-optimized SLAM homologous cDNAs, each with a C-terminal HA tag, were cloned into the lentiviral disease vector CD513B-Cherry using the restriction enzymes *SgrA* I and *Kpn* I. The constructed vector and helper plasmid were co-transfected into 293T cells to package lentivirus. The resulting supernatant was collected and used to transduce new 293T cells. The transduced cells were cultured in DMEM supplemented with 10% FBS and 3.5 μg/mL puromycin, followed by subcloning. One positive clone from each cell line was selected for further subculture.

### Western blot

Cells cultured in 24-well plates were washed twice with sterile PBS and lysed with 80 μL RIPA lysis buffer containing a protease inhibitor (Phenylmethylsulfonyl fluoride (PMSF), Solarbio, 1:100). Following, 20 μL of 5 × SDS loading buffer was added to the lysate, and the mixture was boiled for 10 min. The proteins were then separated by SDS-PAGE using 10% polyacrylamide gels and transferred onto 0.22 µm polyvinylidene difluoride membranes (Millipore, USA). The membranes were blocked with 10% skim milk at room temperature (RT) for 2 h. Subsequently, they were incubated overnight at 4 °C with anti-HA (Cell Signaling Technology, Inc) and anti-β-tubulin (Proteintech, China) primary antibodies. For the infected cell protein samples, additional incubations with anti-GFP and anti-PPRV N protein primary antibodies were performed. The membranes were washed with TBST (TBS buffer containing 0.05% Tween-20) for five times, with each wash lasting for 5 min. The membranes were then incubated with HRP-conjugated secondary antibodies at room temperature for 1 h, followed by washing with TBST five times. Finally, the membranes were exposed and imaged using the ECL Ultra-sensitive Kit (Beijing Dining Biotechnology, China) and a chemiluminescence imaging system.

### Indirect immunofluorescence assay

Cells were seeded in 48-well plates and cultured until confluence reached 60%. The culture medium was then removed, and the cells were washed twice with 100 μL PBS. Cells were fixed with 100 μL of 4% paraformaldehyde (diluted in PBS) for 15 min at RT. Following fixation, cells were permeabilized with 100 μL of 0.2% Triton X-100. Subsequently, cells were blocked with 100 μL PBS containing 1% bovine serum albumin (BSA) at 37 °C for 1 h. Cells were then incubated overnight at 4 °C with HA tag antibody (Cell Signaling Technology, Inc). After five washes with PBS, the cells were incubated with Alexa Fluor 488 goat anti-rabbit secondary antibody (Abcam, UK) at 37 ℃ for 1 h, protected from light, and stained with 4′,6-diamidino-2-phenylindole (DAPI, Sigma, 1:500). Finally, cells were visualized and imaged using a fluorescent microscope.

### PPRV infection

Cells were cultured to approximately 90% confluence in 24-well plates and washed twice with PBS. The virus was diluted in DMEM to achieve multiplicity of infection (MOI) of 1 and added to each well. The inoculated cells were then cultured at 37 °C with 5% CO₂.

### Virus titration and growth curve

Dog SLAM-Vero-Cherry cells were seeded into 96-well plates at a density of 1 × 10^4^ cells/well and cultured overnight. Each well was then inoculated with 100 μL of virus diluted tenfold consecutively. The 50% tissue culture infective dose (TCID_50_) per milliliter was determined using the Reed and Muench method. The growth curve of the virus in cell lines was established by infecting cell monolayers with virus at 1 MOI. Supernatants were collected every 24 h post-inoculation to perform TCID_50_ assays.

### Statistical analysis

Statistical analysis was performed in GraphPad Prism 8 software (GraphPad Software, Inc, La Jolla, CA, USA). The experimental data were analyzed by one-way ANOVA or Student’s *t*-test. Differences were defined statistically significant at *p* < 0.05 (*), *p* < 0.01 (**),* p* < 0.001 (***).

## Results

### Phylogenetic tree of SLAM genes in the ruminant family

Receptors play a crucial role in determining the success of viral infection. Research indicates that the primary receptor for PPRV is SLAM, also known as CD150. While PPRV is known to infect not only goats and sheep but also a range of other ruminants, such as cattle and deer, the capacity of SLAM from ruminants to mediate PPRV infection remains unclear. Additionally, the study of SLAM in ruminants is still limited, with SLAM sequences from most of ruminant species remaining poorly characterized.

**Figure 1:**
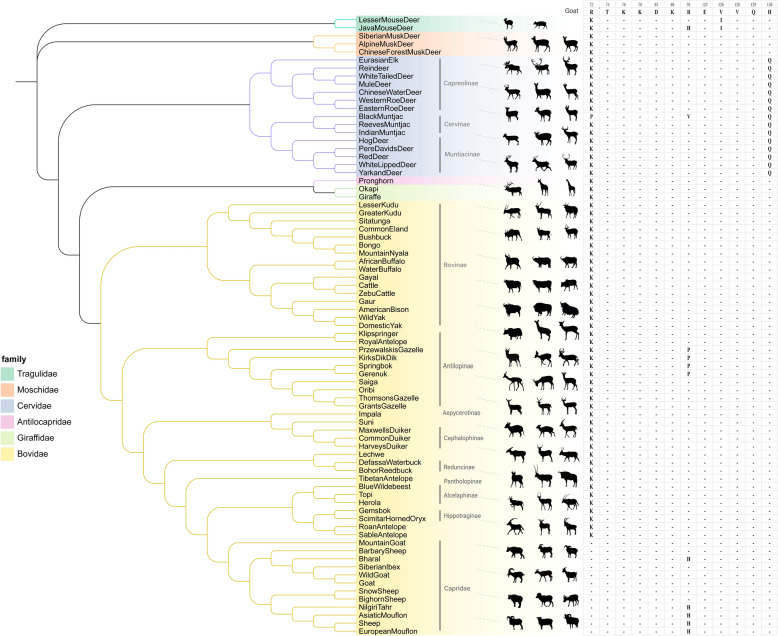
**Genetic and phylogenetic analysis of the SLAM gene from 77 ruminant species.** Analyze the nucleotide sequences of SLAM from the 77 ruminant species and construct a phylogenetic tree based on these sequences. The tree was constructed using MEGA-X software program with the neighbor joining method and bootstrap methods estimated for 1000 replications. The constructed phylogenetic tree divides the ruminants into six families: *Tragulidae*, *Moschidae*, *Cervidae*, *Antilocapridae*, *Giraffidae* and *Bovidae*. The potential PPRV H protein binding domain of SLAMs from 77 ruminant species were showed and compared.

The coding sequence (CDS) region of goat SLAM consists of 1017 nucleotides, encoding 339 amino acids, and locates on chromosome 3 of the goat genome. Analysis comparing the CDS of goat SLAM with the full genome sequence of chromosome 3 reveals that it consists of six discontinuous exons at positions 110924672–110924722, 110926293–110926385, 110933533–110933606, 110937958–110938056, 110952013–110952297, 110954237–110954575 and 110963219–110963294. Based on these alignment results, CDS of SLAM from 76 other ruminant species were successfully extracted from the ruminant whole-genome database [7, 23]. Compared to goat SLAM, the CDS of SLAM from most ruminant species (56 species) also contained 1017 nucleotides. Based on these sequences, a phylogenetic tree of ruminants was constructed, revealing that ruminants were categorized into six families: *Tragulidae*, *Moschidae*, *Cervidae*, *Antilocapridae*, *Giraffidae*, and *Bovidae* (Figure 1)*.* The *Bovidae* family encompassed 54 species, which were further subdivided into nine subfamilies: Bovinae, Antilopinae, Aepycerotinae, Cephalophinae, Reduncinae, Hippotraginae, Alcelaphinae, Pantholopinae, and Caprinae. The *Cervidae* family comprised 15 species, divided into three subfamilies: Capreolinae, Cervinae, and Muntiacinae. The *Moschidae*, *Tragulidae, Giraffidae* and *Antilocapridae* families each contained 1 to 3 species. This SLAM gene-based phylogenetic tree showed significant similarities in the evolutionary relationships among ruminants compared to those derived from whole-genome data [7]. For example, *Antilocapridae* and *Giraffidae* were closely related, while impala and suni formed a sister-group relationship, and bushbuck and mountain nyala were similarly positioned (Figure 1). These findings suggested that SLAM gene similarity among ruminants could partially reflect their evolutionary relationships.

### Analysis of amino acid residues in ruminant SLAMs may interacting with PPRV H protein

**Figure 2:**
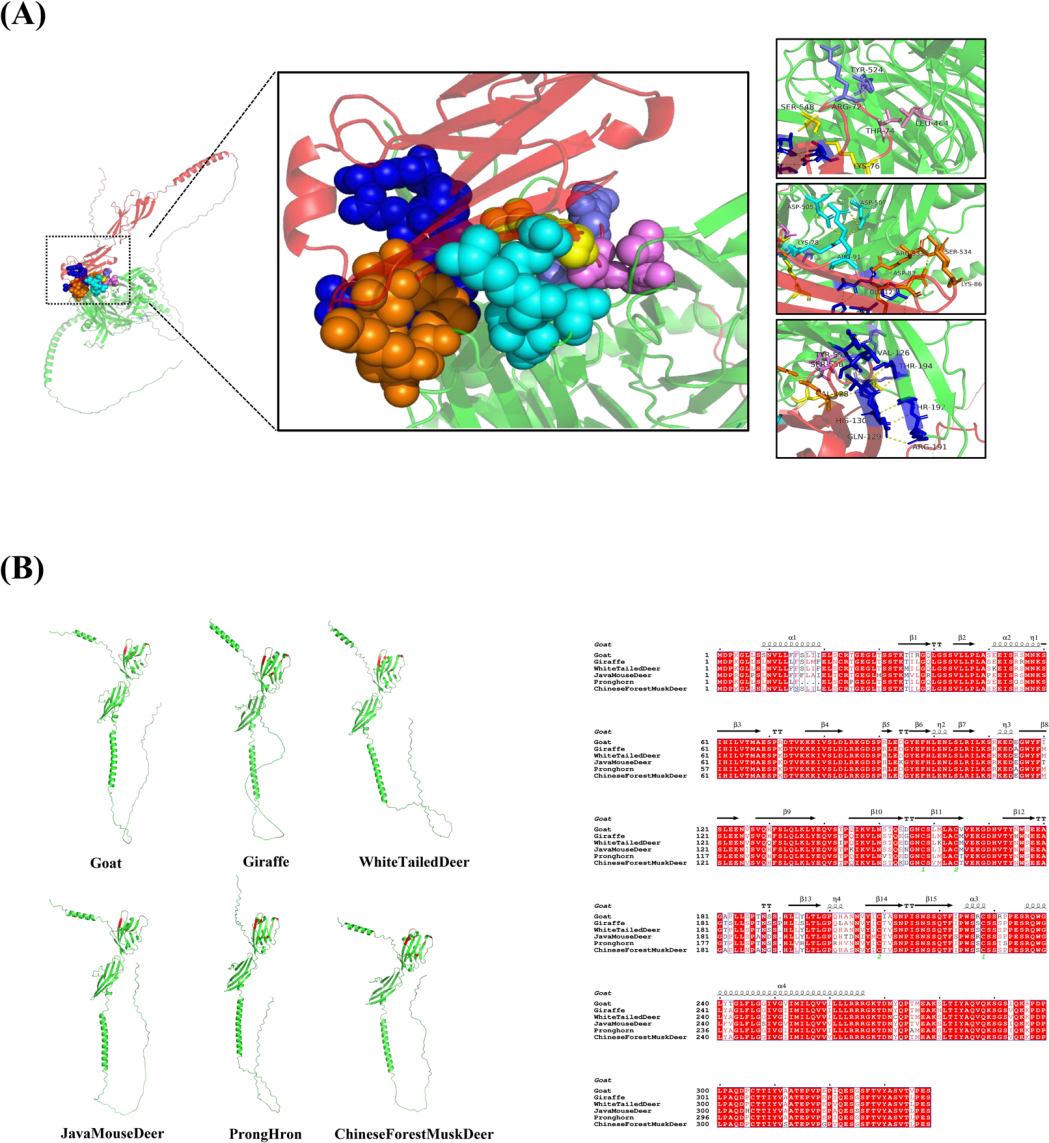
**Structural analysis of the PPRV H-SLAM complex.** **A** Based on the crystal structures of the measles virus H protein and human SLAM protein, a model for the crystal structure of the PPRV H-goat SLAM protein complex was constructed. The resulting crystal structure identifies interactions between PPRV-H and six distinct sites on SLAM. In the figure, sites 1–6 are color-coded as follows: purple, pink, yellow, cyan, orange, and blue. The right panel provides an enlarged view of each site, highlighting the specific amino acid residues involved in the interactions at each location. **B** Based on the crystal structure of human SLAM, models of SLAM from the goat (*Bovidae*), giraffe (*Giraffidae*), white tailed deer (*Cervidae*), java mouse deer (*Tragulidae*), pronghorn (*Antilocapridae*), and Chinese forest musk deer (*Moschidae*)were constructed. The analysis revealed that these models share a similar overall structure. The right side presents the amino acid sequence alignment results for SLAM across six species.

SLAM is a type I transmembrane protein consisting of extracellular, transmembrane, and cytoplasmic regions. The resolved structure of the human SLAM protein shows that the Ig structural domains in the membrane-distal V domain of the extracellular region are primarily recognized by the viral H protein [27, 28]. Based on the crystal structure of the measles virus H protein and human SLAM protein, a model of the PPRV H-goat SLAM protein complex was constructed (PDB ID of the template: 3ALZ. TM-score: 0.52. RMSD^a^: 2.61) [12]. The predicted model revealed that sites on SLAM responsible for interaction were all located in the V region (Figure 2A). Interaction sites 1, 2, and 3 on SLAM, located at ARG72, THR74, and LYS76 on the SLAM β4 sheet, formed hydrogen bonds with PPRV H TYR524, LEU464, and SER548, respectively. Interaction site 4 involved SLAM β4 sheet LYS78 and β5 sheet ARG91, which interacted with PPRV H ASP505, ASP506, ASP507, and ASP530 through hydrogen bonds or salt bridges. Interaction site 5, consisting of SLAM β4 sheet ASP83, LYS86, and β8 sheet GLU123, interacted with PPRV H ARG533 and SER534 via hydrogen bonds, with GLU123 also forming a salt bridge with PPRV H ARG533. Interaction site 6 located on SLAM β9 sheet, including VAL126, VAL128, GLN129, and HIS130, which interacted through hydrogen bonds with PPRV H ARG191, THR192, THR194, SER550, and TYR551.

Based on the predicted interactions with PPRV-H involving 12 amino acids, the comparative analysis was conducted on 77 ruminant SLAM proteins (Figure 1). Most of these amino acids were conserved in ruminant except at positions 72, 91, 126 and 130. At position 72, *Bovidae* subfamily Caprinae had R, while other species, except black muntjac, had K. At position 91, the black muntjac had V, java mouse deer and some members of the *Antilocapridae* subfamily had H, while other species had R. At position 126, *Tragulidae* species had I, while other species had V. At position 130, *Cervidae* ruminants had Q, while other species had H. To further compare SLAM differences among ruminants, representative species were selected from each family: java mouse deer (JMD, *Tragulidae*), giraffe (*Giraffidae*), Chinese forest musk deer (CFMD, *Moschidae*), white tailed deer (WTD, *Cervidae*), pronghorn (PH, *Antilocapridae*), and goat (*Bovidae*). Models for SLAM from these 6 species were constructed based on the crystal structure of human SLAM (Figure 2B, left), revealing a similar overall structural configuration characterized by two β-sheet domains in the extracellular head region. The predicted amino acids interacting with PPRV-H were located in equivalent positions within the V domain across these species. Notably, due to the absence of 4 amino acids at the N-terminus, the predicted pronghorn SLAM lacked an α-helix compared to SLAM from the other 5 species. The extracellular domain included a structure composed of α-helices connected to the head via a disordered linker. However, the transmembrane and intracellular regions were predicted to be disordered due to the lack of a crystal structure reference. Furthermore, a comparison of the amino acid sequences of SLAM from these 6 species (Figure 2B, right) showed overall conservation with only occasional differences in certain regions. For instance, at position 182, goat and Chinese forest musk deer had A, while giraffe, white tailed deer, and pronghorn had T, and java mouse deer had D. These less conserved amino acids did not correspond to the predicted amino acids interacting with the PPRV-H protein. Therefore, we speculated that SLAM from these representative species across the 6 ruminant families may, as goat SLAM, effectively mediate PPRV infection.

### Cell lines expressing ruminant SLAM proteins

**Figure 3:**
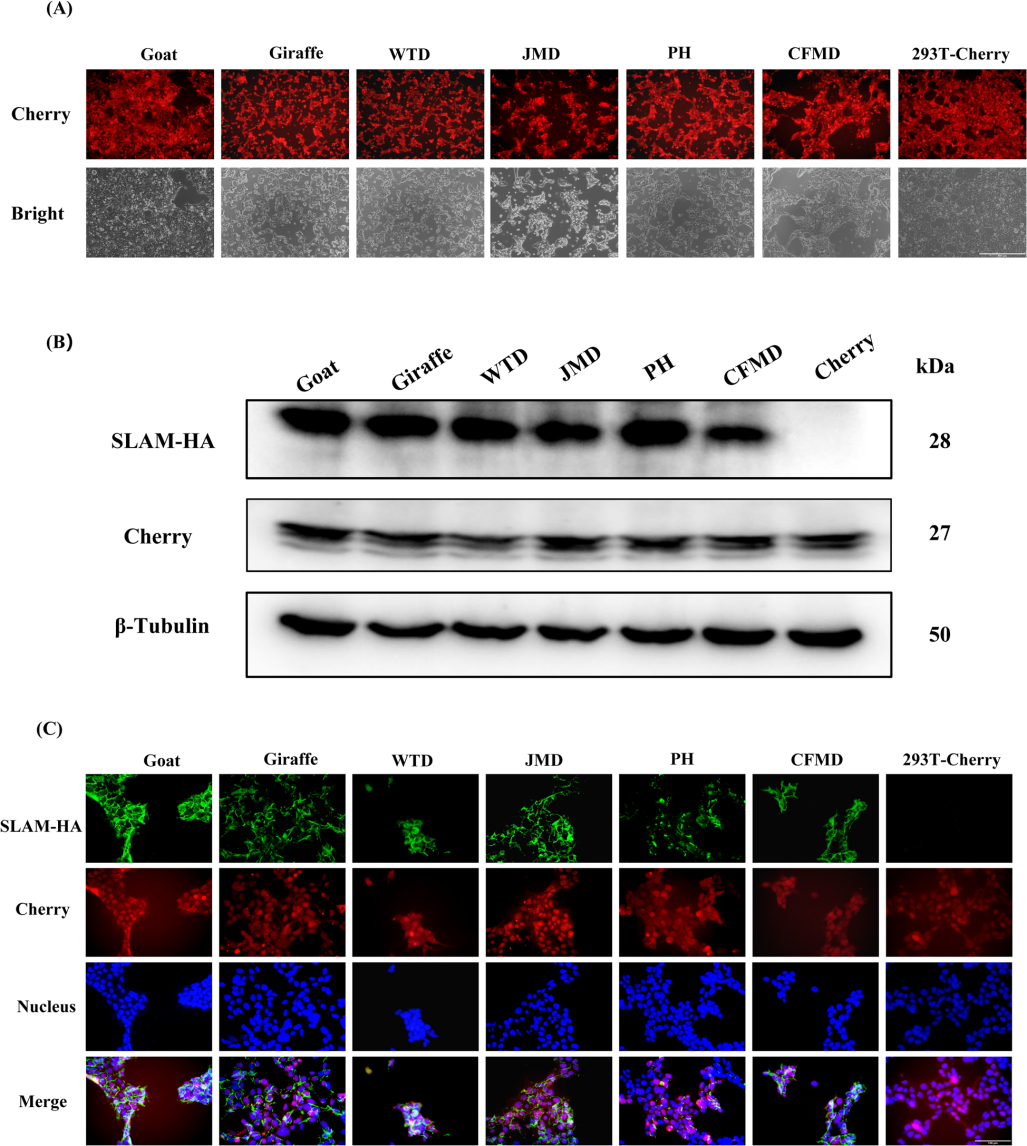
**Construction of cell lines expressing ruminant SLAM protein.** **A** Microscopic examination of the six SLAM cell lines reveals no significant morphological differences compared to SLAM negative 293T-Cherry cells. **B** Western Blot analysis, utilizing the SLAM C-terminal HA tag, confirmed the expression of SLAM in all six cell lines. **C** IFA was performed to evaluate SLAM expression. SLAM-HA was visualized in green, cell nuclei were stained blue, and cells were additionally labeled with cherry protein. The cells were observed using a fluorescent microscope at 400× magnification.

To determine whether SLAM from the six species can mediate PPRV infection, cell lines expressing SLAM were constructed. Previous studies have demonstrated that PPRV is insensitive to 293T cells [29]. Thus, 293T cells were utilized to express SLAM and assess SLAM-mediated PPRV infection. CDS of SLAM from java mouse deer, giraffe, Chinese forest musk deer, white tailed deer, pronghorn, and goat were synthesized, with an HA tag added to the C-terminus and linked to the Cherry via a T2A linker. These sequences were cloned into a lentiviral vector. Following transfection, transduction, drug selection, and subcloning, multiple cell lines expressing the red fluorescent protein were obtained. One cell line per SLAM type was selected for further analysis (Figure 3A). Microscopic observation revealed that the red fluorescent cells adhered to the well, exhibited a polygonal morphology, and grew in patches, similar to wild-type HEK-293T (293T) cells. Upon passaging at a 1:3 ratio, these cells reached approximately 90% confluence within 2–3 days, with a growth rate comparable to wild-type 293T cells. The SLAM gene, theoretically co-expressed with Cherry due to the T2A linkage, was confirmed. The WB showed that all six SLAM-293T cell lines expressed both SLAM and Cherry proteins, whereas the 293T-Cherry cell line only expressed Cherry (Figure 3B). No significant differences in SLAM protein expression levels were observed among the six SLAM-293T cell lines. IFA analysis further confirmed SLAM expression in the SLAM-293T cells, with no SLAM detected in the 293T-Cherry cells (Figure 3C). Additionally, SLAM localization appeared to be at the cell membrane rather than in the nucleus. These results confirmed that these SLAM-expressing cell lines were suitable employed for assessing whether SLAM from different ruminant subfamilies can support PPRV infection.

### Evaluation of SLAMs in mediating PPRV infection

**Figure 4:**
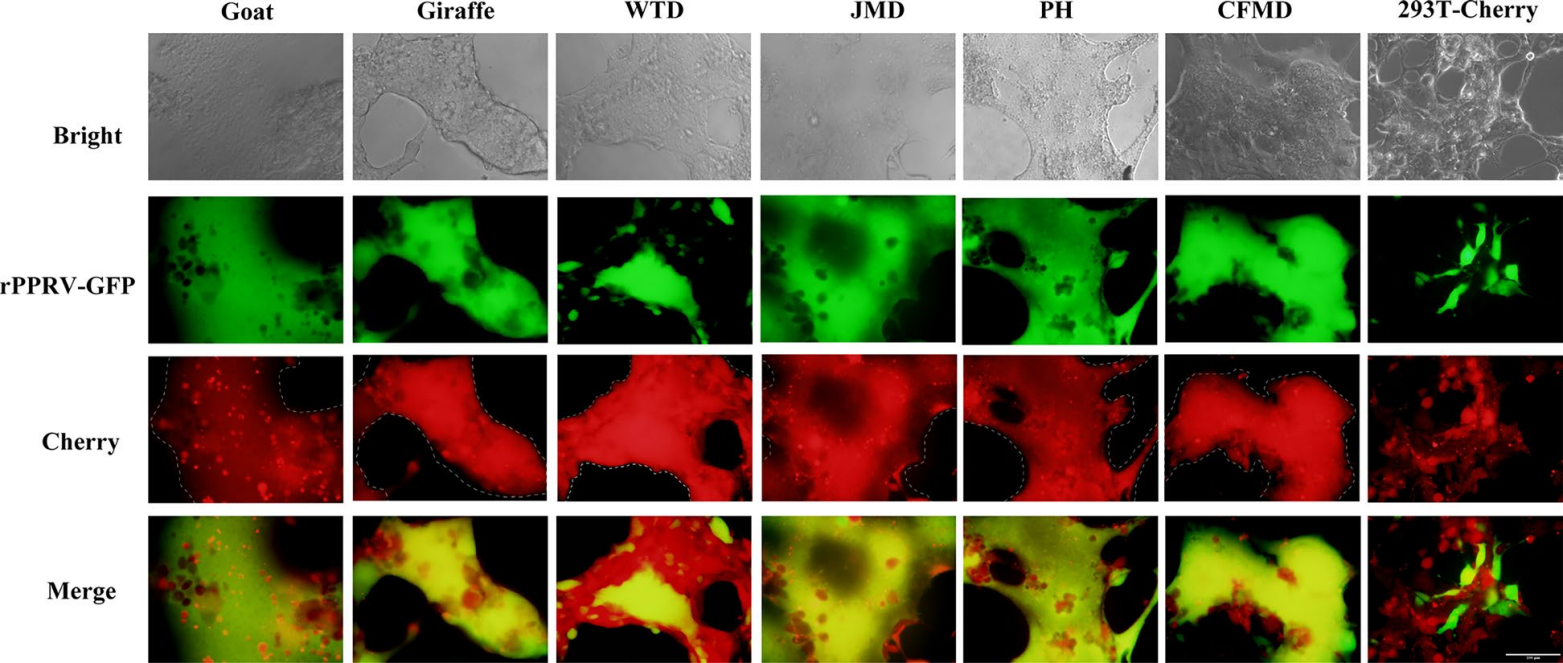
**Syncytia of SLAM-Cells infection with PPRV.** The cells were infected with rPPRV-GFP at an MOI of 2 and incubated for 48 h. Syncytium formation was then assessed using fluorescent microscope, with white dashed lines delineating the contours of the syncytia in the red fluorescece channel.

Previous studies indicated that the goat SLAM not only mediates PPRV infection effectively but also induces cell–cell fusion and syncytium formation [30, 31]. To determine whether other ruminant SLAMS can induce this phenomenon upon PPRV infection, the cells were infected with rPPRV-GFP (PPRV expressing green fluorescent protein (GFP)) and analyzed for syncytium formation (Figure 4). Microscopic observation revealed that 293T-Cherry cells did not form syncytia. In contrast, cells expressing SLAM of java mouse deer, giraffe, Chinese forest musk deer, white tailed deer, pronghorn, or goat exhibited clear syncytium formation. GFP analysis showed that while 293T-Cherry cells were susceptible to viral infection, GFP-positive cells did not form syncytia. Conversely, cells expressing SLAM formed syncytia, with GFP-positive cells participating in this process. Among cells expressing SLAM from java mouse deer, giraffe, Chinese forest musk deer, pronghorn, and goat, syncytia almost exclusively expressed viral GFP. However, cells expressing SLAM from white tailed deer showed inconsistent GFP expression in syncytia.

**Figure 5:**
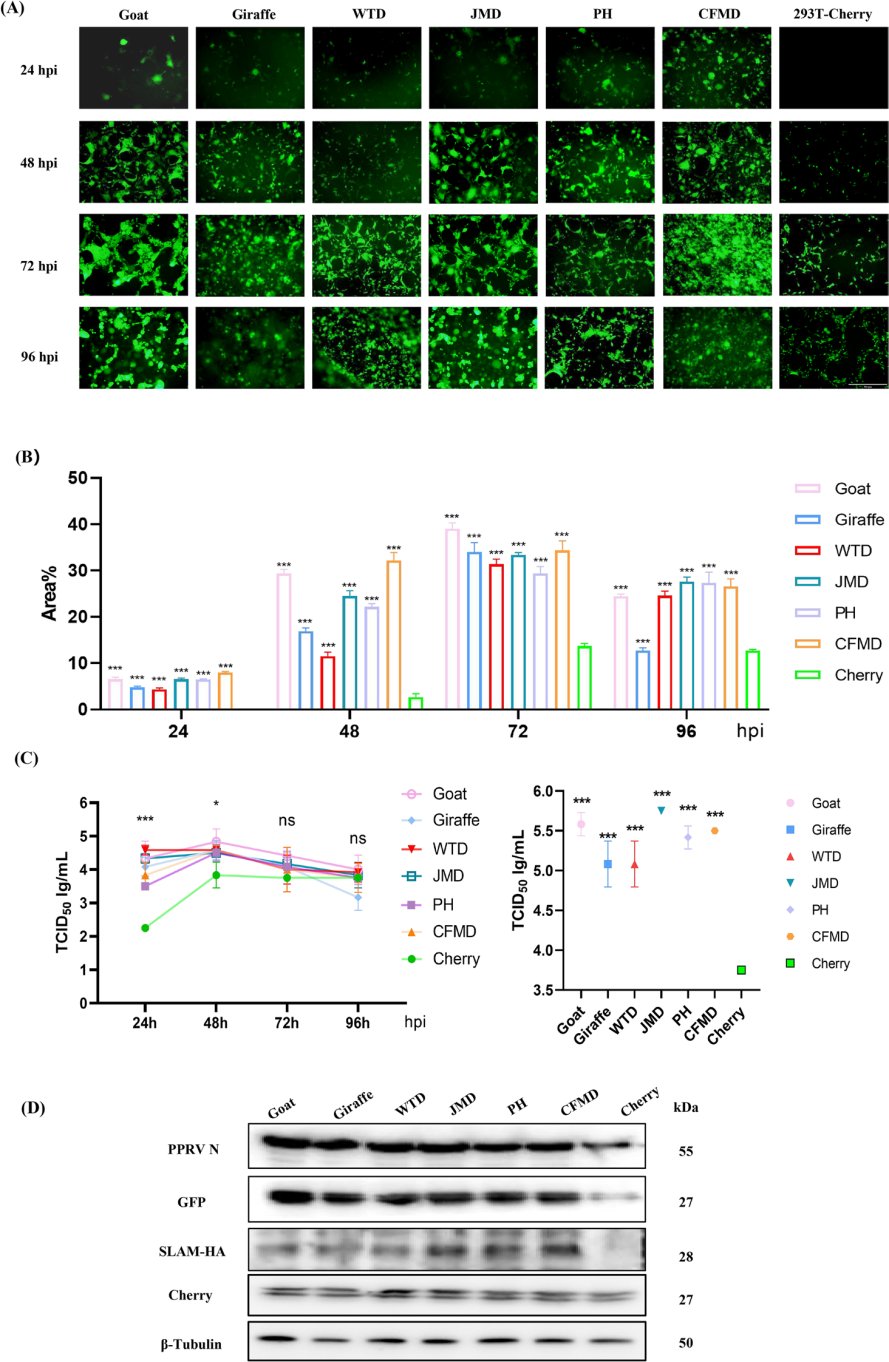
**Replication of PPRV in six SLAM cell lines.** **A** Six SLAM-293T cell lines were infected with rPPRV-GFP at an MOI of 1, and viral GFP protein expression was monitored every 24 h. The 293T-Cherry cell line served as a control. **B** The proportion of green fluorescence area in **A** was quantified and subjected to statistical analysis. Data were shown as the mean ± SD of the results from three individual experiments. *** *p* < 0.001, * *p* < 0.05, ns* p* > 0.05. **C** Multistep growth curves of rPPRV-GFP in six SLAM-expressing 293T cell lines at an MOI of 1. The right panel illustrates the viral titer of rPPRV-GFP following two freeze–thaw cycles at 72^nd^ hpi. Data were shown as the mean ± SD of the results from three individual experiments. *** *p* < 0.001, * *p* < 0.05, ns* p* > 0.05. **D** Cellular protein samples were collected at 72^nd^ hpi to detect viral protein expression.

To assess whether SLAM expression enhances viral replication, we analyzed GFP expression following rPPRV-GFP infection at various time points using fluorescent microscopy (Figure 5A). At the 24^th^ hpi, SLAM-expressing cells exhibited weak green fluorescence and minimal syncytia formation. Among these, 293T-Cherry-CFMD cells demonstrated the most extensive syncytia formation, while 293T-Cherry cells did not show green fluorescence. At the 48^th^ and 72^nd^ hpi, fluorescent intensity increased across all six SLAM-expressing cell lines, with syncytia enlarging. Due to virus-induced cytopathic effects (CPE) leading to cell death, fluorescent intensity decreased at the 96^th^ hpi. Although 293T-Cherry cells began to show green fluorescence at the 48^th^ hpi, the fluorescent area ratio was significantly lower than in the six SLAM-expressing cell lines, and no prominent syncytia were observed (Figure 5B). Additionally, to compare viral replication across various cell lines, cell supernatants were collected at each time point and measured viral titers (Figure 5C). At the 24^th^ hpi, viral titers in the supernatants from six SLAM-expressing cell lines exceeded 10^3.5^ TCID_50_/mL, which was 17 to 177 times higher than the titer in supernatants from 293T-Cherry cells. The virus continued to amplify in all cell lines. However, the difference in viral titer between SLAM-expressing cell lines and the 293T-Cherry cells narrowed to approximately tenfold at the 48^th^ hpi. Then, viral titers in the cell supernatants decreased and showed no significant difference compared to the 293T-Cherry control group from the 72^nd^ to 96^th^ hpi.

The discrepancy in viral titer and fluorescence might be due to a significant amount of viral particles remaining intracellularly and not being released into supernatants. To address this, we subjected the cells to two freeze–thaw cycles at the 72^nd^ hpi to release intracellular virus and re-measured viral titers (Figure 5C). The viral titer in 293T-Cherry cells was 10^3.75^ TCID_50_/mL, similar to the titer in the supernatant. In contrast, viral titers in SLAM-expressing cell lines exceeded 10^5^ TCID_50_/mL, with 293T-Cherry-JMD cells reaching a titer of 10^5.75^ TCID_50_/mL, 100 times higher than that in 293T-Cherry cells. WB analysis of cell proteins at the 72^nd^ hpi revealed substantial levels of GFP and NP proteins from rPPRV-GFP in SLAM-expressing 293T cells (Figure 5D). Although there were no significant differences in viral protein expression among the SLAM-expressing cells, the levels were notably higher than in 293T-Cherry cells. These results confirmed that SLAM from different ruminant species can effectively mediate PPRV infection, which suggest that these species could be potential hosts for PPRV.

## Discussion

Since its initial discovery in Côte d’Ivoire in the early 1940s, PPRV has progressively expanded its geographical distribution to approximately 70 countries across Africa, the Middle East, and parts of Asia over the past 20 years [32]. Its high pathogenicity and mortality rate have exerted severe socio-economic impacts on livestock industries in many countries [33]. Despite the extensive use of effective attenuated vaccines to protect sheep and goats from PPRV, the virus remains endemic in certain regions of the world [34, 35]. In recent decades, there has been a rising incidence of PPRV infections in both domestic and wild animals not traditionally recognized as natural hosts, such as dromedaries, gazelles, wild goats, golden jackals, deer, antelopes, wild boars, and pigs [21, 36–39]. This observation suggests that PPRV may possess the ability to adapt to new hosts, thereby expanding its host range. Such dynamics present significant challenges to global efforts aimed at controlling and eradicating PPRV.

The virus receptor interaction is a major determinant of the cell tropism. Research has identified SLAM as the primary receptor for PPRV. Although studies have shown that SLAM from goats, sheep, cattle, and buffalo share high homology and that key amino acids in the V region are highly conserved, research on SLAM from other ruminants is limited, and it remains unclear whether these receptors can mediate viral infection [18]. This gap in knowledge restricts our understanding of potential PPRV hosts. To address this, we analyzed SLAM proteins from various ruminants to predict potential PPRV hosts by examining their phylogenetic relationships. We extracted CDS sequences of SLAM from 76 ruminant species, excluding goats, from a ruminant genomic library and constructed a phylogenetic tree based on nucleotide sequences (Figure 1). We constructed the phylogenetic tree using the Neighbor-Joining (NJ) method, which is commonly used for building phylogenetic trees, especially when handling large sequence datasets. It effectively processes distance matrices and generates the optimal tree structure through iterative optimization [40]. The phylogenetic tree derived from SLAM gene sequences was highly similar to that based on whole-genome data, although some differences were observed. For example, while suni is classified under the Antilopinae subfamily of *Bovidae* in whole-genome analyses, its SLAM was found to be more closely related to the Cephalophinae subfamily [7]. Among the 77 ruminants analyzed, the CDS sequences of SLAM in most species (56) consisted of 1017 nucleotides, identical to that of goats. In contrast, giraffes and okapis exhibited three additional nucleotides, leading to an extra amino acid at the position 192 of the SLAM protein. Furthermore, 18 species displayed nucleotide deletions at various positions in their SLAM sequences. Unfortunately, it has not yet been determined whether these differences in nucleotide length are due to sequencing errors or other factors. If these sequences are correct, these species will express abnormal SLAM which may not interact with PPRV H protein.

The crystal structure of the MeV H protein and the SLAMF1-V domain complex, as described by Hashiguchi et al., reveals that the β4-6 region of the H protein interacts with the GFCCC region of the SLAMF1-V domain [41]. Critical for stabilizing this complex are the salt bridges between residues Asp530 and Arg533 of the H protein and Glu123 of the SLAMF1-V domain. Additionally, intermolecular β-sheet interactions between residues Pro191-Arg196 (in β6) of the H protein and residues Ser127-Phe131 of SLAMF1-V further stabilize the complex [12, 42]. Utilizing the crystal structures of the MeV H protein and human SLAM protein, we have predicted the crystal structure of the goat SLAM-PPRV H complex (Figure 2A) and analyzed the 12 amino acids of SLAM interacting with PPRV H. Comparative analysis of SLAM proteins from 77 ruminant species (Figure 1) identified 8 highly conserved amino acids, including the amino acid at position 123, which is Glu in all ruminant SLAM proteins. This suggests that SLAM proteins across these ruminant species may have the capability to bind PPRV H. In goat SLAM, four amino acid residues—VAL126, VAL128, GLN 129, and HIS130—contribute to the stabilization of the PPRV H-SLAM complex. VAL128 and GLN129 are conserved across ruminant species, whereas the residue at position126 is Ile in Tragulidae and Val in the other five families of ruminants. For residue 130, Cervidae possess Gln, while the other five families have His. Further investigation is required to determine whether these amino acid variations affect the efficiency of SLAM binding to PPRV H. This will involve constructing and analyzing cell lines to validate these findings. During the entry of the virus into the host cell, F protein attaches to the membrane and HRA (heptad repeat A) and HRB (heptad repeat B) interact with each other, resulting in fusion of the virus and host by bringing them close to each other [14]. Therefore, the F protein also plays an important role in the process of PPRV infection in cells. The interaction between the F protein and host cells should be considered in a comprehensive evaluation of PPRV’s infectivity. Unfortunately, we have not yet conducted an in-depth study of this aspect.

We selected a representative species from six different families and predicted the crystal structures of their SLAM proteins. Subsequently, we established cell lines expressing SLAM from these six species. The studies demonstrated that SLAM from these species can effectively mediate PPRV infection. The absence of the N-terminal α-helix in pronghorn SLAM did not impact its ability to mediate PPRV infection, suggesting that these species may serve as potential hosts for PPRV (Figure 5). Additionally, the evaluation of PPRV proliferation in JMD-SLAM-293T cells showed the highest efficiency (Figure 5B), indicating that this cell line could be utilized for large-scale virus propagation in vaccine generation. Analysis of syncytia formation and viral titers post-PPRV infection revealed that, compared to other SLAMs, SLAM of white tailed deer exhibited less infection efficiency (Figure 4). Based on the crystal structure analysis of the goat SLAM-PPRV H complex, we hypothesize that this reduced efficiency may be attributed to the Gln residue at position 130 in *Cervidae* SLAM, as opposed to His in other species. For human SLAM, the 130th amino acid is His and plays a pivotal role for interaction with Mev H protein [12, 43].

In addition to pronghorn, sequencing results from SLAM genes of various other species also reveal nucleotide deletions. However, we have not yet synthesized these genes to construct stable cell lines for assessing their ability to mediate PPRV infection. Furthermore, it remains undetermined whether these nucleotide length discrepancies are due to sequencing assembly errors. Should sequencing confirm that these nucleotide deletions are authentic, they may lead to frame shifts during translation, potentially resulting in improper protein expression (see Additional file 1). Future research will involve cloning these SLAM genes into lentiviral vectors to develop stable cell lines, which will be used to evaluate their ability to mediate PPRV infection. If it is confirmed that nucleotide deletions in SLAM genes from these species contribute to reduced susceptibility to PPRV, it would suggest that not all ruminants are susceptible to the virus. This finding would provide significant insights for the development of ruminant species that are less susceptible to PPRV.

In summary, we conducted a preliminary analysis of SLAM receptors from 77 ruminant species and selected representative species to evaluate their capacity to mediate PPRV infection. Both genetic evolutionary analysis and SLAM functional validation indicate that these species are capable of mediating PPRV infection, thereby providing deeper insights into the potential host range of this virus at the cellular level. The results also suggest that the distribution of PPRV may be significantly broader than previously recognized, underscoring the necessity of monitoring susceptible hosts and contributing positively to the global effort to eradicate PPRV.

In this study, we analyzed SLAM receptors from 77 ruminant species and identified them as highly conserved, particularly in regions likely involved in interactions with the viral H protein. Additionally, cells expressing SLAM from each ruminant family effectively mediated PPRV infection and replication. These findings suggested that SLAM receptors from various ruminant species can facilitate PPRV infection, indicating that PPRV might have the potential to infect many ruminants. Consequently, these potential host populations should be considered in strategies aimed at preventing and eradicating PPRV.

## Supplementary Information

**Additional file 1. 77 kinds of ruminant SLAM amino acid sequences. **Based on the positioning of the SLAM gene on chromosome 3 in goats, exonic sequences of SLAM genes from 76 additional ruminant species were retrieved from the ruminant genome database. These 77 SLAM exonic sequences were subsequently translated to generate the corresponding amino acid sequences.

## Data Availability

The datasets used and/or analyzed during the current study are available from the corresponding author on reasonable request.

## References

[1] Barrett T, Amarel-Doel C, Kitching RP, Gusev A (1993) Use of the polymerase chain reaction in differentiating rinderpest field virus and vaccine virus in the same animals. Rev Sci Tech 12:865–872 8219336 10.20506/rst.12.3.734

[2] Bailey D, Banyard A, Dash P, Ozkul A, Barrett T (2005) Full genome sequence of peste des petits ruminants virus, a member of the *Morbillivirus* genus. Virus Res 110:119–124 15845262 10.1016/j.virusres.2005.01.013

[3] Zhu Z, Zhang X, Adili G, Huang J, Du X, Zhang X, Li P, Zheng X, Liu X, Zheng H, Xue Q (2016) Genetic characterization of a novel mutant of peste des petits ruminants virus isolated from Capra ibex in China during 2015. Biomed Res Int 2016:7632769 26998489 10.1155/2016/7632769 PMC4779526

[4] Mao L, Li W, Hao F, Yang L, Li J, Sun M, Zhang W, Liu M, Luo X, Cheng Z (2022) Research progress on emerging viral pathogens of small ruminants in China during the last decade. Viruses 14:1288 35746759 10.3390/v14061288 PMC9228844

[5] Schmitz KS, Eblé PL, van Gennip RGP, Maris-Veldhuis MA, de Vries RD, van Keulen LJM, de Swart RL, van Rijn PA (2023) Pathogenesis of wild-type- and vaccine-based recombinant peste des petits ruminants virus (PPRV) expressing EGFP in experimentally infected domestic goats. J Gen Virol 104:001828 10.1099/jgv.0.001828 36757863

[6] Baron MD, Diop B, Njeumi F, Willett BJ, Bailey D (2017) Future research to underpin successful peste des petits ruminants virus (PPRV) eradication. J Gen Virol 98:2635–2644 29022862 10.1099/jgv.0.000944 PMC5845661

[7] Chen L, Qiu Q, Jiang Y, Wang K, Lin Z, Li Z, Bibi F, Yang Y, Wang J, Nie W, Su W, Liu G, Li Q, Fu W, Pan X, Liu C, Yang J, Zhang C, Yin Y, Wang Y, Zhao Y, Zhang C, Wang Z, Qin Y, Liu W, Wang B, Ren Y, Zhang R, Zeng Y, da Fonseca RR, Wei B, Li R, Wan W, Zhao R, Zhu W, Wang Y, Duan S, Gao Y, Zhang YE, Chen C, Hvilsom C, Epps CW, Chemnick LG, Dong Y, Mirarab S, Siegismund HR, Ryder OA, Gilbert MTP, Lewin HA, Zhang G, Heller R, Wang W (2019) Large-scale ruminant genome sequencing provides insights into their evolution and distinct traits. Science 364:eaav6202 31221828 10.1126/science.aav6202

[8] Wernike K, Eschbaumer M, Breithaupt A, Maltzan J, Wiesner H, Beer M, Hoffmann B (2014) Experimental infection of sheep and goats with a recent isolate of peste des petits ruminants virus from Kurdistan. Vet Microbiol 172:140–145 24908276 10.1016/j.vetmic.2014.05.010

[9] Pruvot M, Fine AE, Hollinger C, Strindberg S, Damdinjav B, Buuveibaatar B, Chimeddorj B, Bayandonoi G, Khishgee B, Sandag B, Narmandakh J, Jargalsaikhan T, Bataa B, McAloose D, Shatar M, Basan G, Mahapatra M, Selvaraj M, Parida S, Njeumi F, Kock R, Shiilegdamba E (2020) Outbreak of peste des petits ruminants among critically endangered Mongolian Saiga and other wild ungulates, Mongolia, 2016–2017. Emerg Infect Dis 26:51–62 31855146 10.3201/eid2601.181998 PMC6924898

[10] Govindarajan R, Koteeswaran A, Venugopalan AT, Shyam G, Shaouna S, Shaila MS, Ramachandran S (1997) Isolation of pestes des petits ruminants virus from an outbreak in Indian buffalo (*Bubalus bubalis*). Vet Rec 141:573–574 9423239 10.1136/vr.141.22.573

[11] Zakian A, Nouri M, Kahroba H, Mohammadian B, Mokhber-Dezfouli MR (2016) The first report of peste des petits ruminants (PPR) in camels (*Camelus dromedarius*) in Iran. Trop Anim Health Prod 48:1215–1219 27155951 10.1007/s11250-016-1078-6

[12] Lin LT, Richardson CD (2016) The host cell receptors for measles virus and their interaction with the viral hemagglutinin (H) protein. Viruses 8:250 27657109 10.3390/v8090250 PMC5035964

[13] Plattet P, Plemper RK (2013) Envelope protein dynamics in paramyxovirus entry. MBio 4:e00413-13 23820396 10.1128/mBio.00413-13 PMC3705453

[14] Prajapati M, Alfred N, Dou Y, Yin X, Prajapati R, Li Y, Zhang Z (2019) Host cellular receptors for the peste des petits ruminant virus. Viruses 11:729 31398809 10.3390/v11080729 PMC6723671

[15] Birch J, Juleff N, Heaton MP, Kalbfleisch T, Kijas J, Bailey D (2013) Characterization of ovine Nectin-4, a novel peste des petits ruminants virus receptor. J Virol 87:4756–4761 23388720 10.1128/JVI.02792-12 PMC3624396

[16] Delpeut S, Noyce RS, Richardson CD (2014) The V domain of dog PVRL4 (nectin-4) mediates canine distemper virus entry and virus cell-to-cell spread. Virology 454–455:109–117 24725937 10.1016/j.virol.2014.02.014

[17] Chen Y, Wang T, Yang Y, Fang Y, Zhao B, Zeng W, Lv D, Zhang L, Zhang Y, Xue Q, Chen X, Wang J, Qi X (2022) Extracellular vesicles derived from PPRV-infected cells enhance signaling lymphocyte activation molecular (SLAM) receptor expression and facilitate virus infection. PLoS Pathog 18:e1010759 36084159 10.1371/journal.ppat.1010759 PMC9491601

[18] Sarkar J, Balamurugan V, Sen A, Saravanan P, Sahay B, Rajak KK, Rasool TJ, Bhanuprakash V, Singh RK (2009) Sequence analysis of morbillivirus CD150 receptor-Signaling Lymphocyte Activation Molecule (SLAM) of different animal species. Virus Genes 39:335–341 19669672 10.1007/s11262-009-0391-9

[19] Ohishi K, Ando A, Suzuki R, Takishita K, Kawato M, Katsumata E, Ohtsu D, Okutsu K, Tokutake K, Miyahara H, Nakamura H, Murayama T, Maruyama T (2010) Host-virus specificity of morbilliviruses predicted by structural modeling of the marine mammal SLAM, a receptor. Comp Immunol Microbiol Infect Dis 33:227–241 19027953 10.1016/j.cimid.2008.10.003

[20] Balamurugan V, Krishnamoorthy P, Veeregowda BM, Sen A, Rajak KK, Bhanuprakash V, Gajendragad MR, Prabhudas K (2012) Seroprevalence of Peste des petits ruminants in cattle and buffaloes from Southern Peninsular India. Trop Anim Health Prod 44:301–306 22105906 10.1007/s11250-011-0020-1

[21] Munir M (2014) Role of wild small ruminants in the epidemiology of peste des petits ruminants. Transbound Emerg Dis 61:411–424 23305511 10.1111/tbed.12052

[22] Hu Q, Chen W, Huang K, Baron MD, Bu Z (2012) Rescue of recombinant peste des petits ruminants virus: creation of a GFP-expressing virus and application in rapid virus neutralization test. Vet Res 43:48 22658079 10.1186/1297-9716-43-48 PMC3412694

[23] The Ruminant Genome Database. http://animal.omics.pro/code/index.php/RGD. Accessed 06 Nov 2023.

[24] Xie J, Chen Y, Cai G, Cai R, Hu Z, Wang H (2023) Tree visualization By One Table (tvBOT): a web application for visualizing, modifying and annotating phylogenetic trees. Nucleic Acids Res 51:W587–W592 37144476 10.1093/nar/gkad359 PMC10320113

[25] Robert X, Gouet P (2014) Deciphering key features in protein structures with the new ENDscript server. Nucleic Acids Res 42:W320–W324 24753421 10.1093/nar/gku316 PMC4086106

[26] Zheng W, Wuyun Q, Li Y, Zhang C, Freddolino PL, Zhang Y (2024) Improving deep learning protein monomer and complex structure prediction using DeepMSA2 with huge metagenomics data. Nat Methods 21:279–289 38167654 10.1038/s41592-023-02130-4 PMC10864179

[27] van Driel BJ, Liao G, Engel P, Terhorst C (2016) Responses to microbial challenges by SLAMF receptors. Front Immunol 7:4 26834746 10.3389/fimmu.2016.00004 PMC4718992

[28] Veillette A, Dong Z, Latour S (2007) Consequence of the SLAM-SAP signaling pathway in innate-like and conventional lymphocytes. Immunity 27:698–710 18031694 10.1016/j.immuni.2007.11.005

[29] Liu F, Zhang Y, Li L, Zuo Y, Sun C, Xiaodong W, Wang Z (2019) Rescue of eGFP-expressing small ruminant morbillivirus for identifying susceptibilities of eight mammalian cell lines to its infection. Virus Res 261:60–64 30578803 10.1016/j.virusres.2018.12.011

[30] Adombi CM, Lelenta M, Lamien CE, Shamaki D, Koffi YM, Traoré A, Silber R, Couacy-Hymann E, Bodjo SC, Djaman JA, Luckins AG, Diallo A (2011) Monkey CV1 cell line expressing the sheep-goat SLAM protein: a highly sensitive cell line for the isolation of peste des petits ruminants virus from pathological specimens. J Virol Methods 173:306–313 21371505 10.1016/j.jviromet.2011.02.024 PMC3166437

[31] Qi X, Wang T, Li Z, Wan Y, Yang B, Zeng W, Zhang Y, Wang J (2019) MicroRNA-218 regulates Signaling Lymphocyte Activation Molecular (SLAM) mediated peste des petits ruminants virus infectivity in goat peripheral blood mononuclear cells. Front Immunol 10:2201 31616415 10.3389/fimmu.2019.02201 PMC6763950

[32] Rume VN, Dundon WG, Belay G, Baziki JD, Diakite A, Paul A, Tessema YD, Nwankpa N, Gizaw D, Cattoli G, Bodjo SC, Tessema TS (2019) Molecular epidemiological update of Peste des Petits Ruminants virus (PPRV) in Ethiopia. Vet Microbiol 235:229–233 31383306 10.1016/j.vetmic.2019.07.006

[33] Dou Y, Liang Z, Prajapati M, Zhang R, Li Y, Zhang Z (2020) Expanding diversity of susceptible hosts in peste des petits ruminants virus infection and its potential mechanism beyond. Front Vet Sci 7:66 32181263 10.3389/fvets.2020.00066 PMC7059747

[34] Hodgson S, Moffat K, Hill H, Flannery JT, Graham SP, Baron MD, Darpel KE (2018) Comparison of the immunogenicities and cross-lineage efficacies of live attenuated peste des petits ruminants virus vaccines PPRV/Nigeria/75/1 and PPRV/Sungri/96. J Virol 92:e01471–18 30258008 10.1128/JVI.01471-18 PMC6258957

[35] Kumar N, Barua S, Riyesh T, Tripathi BN (2017) Advances in peste des petits ruminants vaccines. Vet Microbiol 206:91–101 28161212 10.1016/j.vetmic.2017.01.010 PMC7130925

[36] Benfield CTO, Hill S, Shatar M, Shiilegdamba E, Damdinjav B, Fine A, Willett B, Kock R, Bataille A (2021) Molecular epidemiology of peste des petits ruminants virus emergence in critically endangered Mongolian saiga antelope and other wild ungulates. Virus Evol 7:veab062 34754511 10.1093/ve/veab062 PMC8570150

[37] Fakri FZ, Bamouh Z, Jazouli M, Omari Tadlaoui K, Elharrak M (2019) Experimental infection of dromedary camels with virulent virus of Peste des Petits Ruminants. Vet Microbiol 235:195–198 31383302 10.1016/j.vetmic.2019.07.004

[38] Khalafalla AI, Saeed IK, Ali YH, Abdurrahman MB, Kwiatek O, Libeau G, Obeida AA, Abbas Z (2010) An outbreak of peste des petits ruminants (PPR) in camels in the Sudan. Acta Trop 116:161–165 20707980 10.1016/j.actatropica.2010.08.002

[39] Schulz C, Fast C, Schlottau K, Hoffmann B, Beer M (2018) Neglected hosts of small ruminant morbillivirus. Emerg Infect Dis 24:2334–2337 30457523 10.3201/eid2412.180507 PMC6256395

[40] Pavlopoulos GA, Soldatos TG, Barbosa-Silva A, Schneider R (2010) A reference guide for tree analysis and visualization. BioData Min 3:1 20175922 10.1186/1756-0381-3-1 PMC2844399

[41] Hashiguchi T, Ose T, Kubota M, Maita N, Kamishikiryo J, Maenaka K, Yanagi Y (2011) Structure of the measles virus hemagglutinin bound to its cellular receptor SLAM. Nat Struct Mol Biol 18:135–141 21217702 10.1038/nsmb.1969

[42] Navaratnarajah CK, Vongpunsawad S, Oezguen N, Stehle T, Braun W, Hashiguchi T, Maenaka K, Yanagi Y, Cattaneo R (2008) Dynamic interaction of the measles virus hemagglutinin with its receptor signaling lymphocytic activation molecule (SLAM, CD150). Biol Chem 283:11763–11771 10.1074/jbc.M800896200 PMC2431048 18292085

[43] Meng X, Zhu X, Alfred N, Zhang Z (2020) Identification of amino acid residues involved in the interaction between *peste-des-petits-ruminants* virus haemagglutinin protein and cellular receptors. J Gen Virol 101:242–251 31859612 10.1099/jgv.0.001368 PMC7416607

