# Supplementary material for: Genetic analyses and functional validation of ruminant SLAMs reveal potential hosts for PPRV
Source: Vet Res. 2025 Mar 18;56:57. doi: 10.1186/s13567-025-01489-w (PMC11916873; doi:10.1186/s13567-025-01489-w)
Supplement: Supplementary file 1 — Additional file 1. 77 kinds of ruminant SLAM amino acid sequences. Based on the positioning of the SLAM gene on chromosome 3 in goats, exonic sequences of SLAM genes from 76 additional ruminant species were retrieved from the ruminant genome database. These 77 SLAM exonic sequences were subsequently translated to generate the corresponding amino acid sequences. [file 13567_2025_1489_MOESM1_ESM.docx]

>Goat

MDPKGLLSSNVLLFFSLIIELSCRTGEGLTSSTKTIRGQLGSSVLLPLASEEISRSMNKSIHILVTMAESPRDTVKKKIVSLDLRKGDSPRLEDGYEFHLENLSLRILKSRKEDEGWYFISLEENVSVQHFSLQLKLYEQVSTPQIKVLNSTQEDGNCSLMLACVVEKGDHVTYNWSEEAGAPLLSPTNSSHLLYLTLGPQHANNVYICIASNPISNSSQTFIPWSRCSSRPPESRQWGLYTGLFLGGIVGVIMILQVVILLLRRRGKTDNYQPTMEAKSLTIYAQVQKSGSIQKRPDPLPAQDPCTTIYVAATEPVPEPIQESGSFTVYASVTVPES*

>LesserMouseDeer

MDPRGLPSLNVLLFFFLAIELTCRTGEGLMSSTKMVLGRLGSSVLLPLAPEEISRSMNKSIHILVTMAESPKDTVKKKIVSLDLRKGDSPRGERL*VSSGKPEPEDPEEQEGR*GLVLYVSGGKHFCPAL*LTAEAL*AGFNSENQGVELQSGKRKLQPDTGLYGGEGGPCDLQLE*GSR*PTAEPCQ*LPPPVSHPWPSAY*QHLHVHCEQSHQQQLSDIQPMVQMQLQSSRIKAMGSVCWTLLREHRWCHHDSPSGGTTVEKKR*NRQLPANSGSKKPYYLCPSSEIRFYSEET*SPASSGPLHDHLRRCHRACPRACPGIKLLHGLCQCDTSREL

>Giraffe

MDPKGLLSLNVLLLFSLMFELNCRTGEGLTSSTKTILGQLGSSVLLPLASEEISRRMNKSIHILVTMAESPKDTVKKKIVSLDLRKGDSPRLEDGYEFHLENLSLRILKSRKEDAGWYFMSLEENVSVQHFSLQLKLYEQVSTPQIKVLNSTQEGGNCSLMLACMVEKGDHVTYHWNEEAGTSLLSPTNSSPHLLSLTLGPQLANNVYICTVSNPISNSSQTFIPWSSCSSSPPESRQWGLYAGLFLGGIVGIIMILQVVILLLRRRGKTDNYQPTMEAKNLTIYAQVQKSGSVQKKPDPLPAQDPCTTIYVAATEPVPEPIQESGSFTVYASVTLPES*

>Reindeer

MDPKGLLSVNVLLFSSLIFELSCRTGEGLTSSTKMILGQLGSSVLLPLASKEISRSMNKSIHILVTMAESPKDTVKKKIVSLDLRKGDSPRLEDGYEFHLENLSLRILKSKKEDEGWYFMSLEENVSVQQFSLQLKLYEQVSIPQIKVLNSTQEDGNCSLKLACMVEKGDHVTYNWSEEAGTPLLSPANSSHLLYLTLGPQHANNVYICTVSNPISNSSQTFIPWSSCSSSPPESRQWGLYAGLFLGGIVGIIMILQVVILLLRRRGKTDNYQPTMEAKSLTIYAQVQKSGSVQKKPDPLPAQDPCTTIYVAATEPVPEPVQESGSFTVYASVTLPES*

>WesternRoeDeer

MDPKGLLSLNVLLFFSLIFELSCRTGEGLTSSTKTILGQLGSSVLLPLASKEISRSMNKSIHILVTMAESPKDTVKKKIVSLDLRKGDSPRLEDGYEFHLENLSLRILKSKKEDEGWYFMSLEENVSVQQFSLQLKLYEQVSIPQIKVLNSTQEDGNCSLKLACMVEKGDHVTYNWSEEAGTPLLSPANSSHLLYLTLGPQHANNVYICTVSNPISNSSQTFIPWSSCSSSPPESRQWGLYAGLFLGGIVGIIMILQVVILLLRRRGKTDNYQPTMEAKSLTIYAQVQKSGSVQKKPDPLPAQDPCTTIYVAATEPVPEPIQESGSFTVYASVTLPES*

>WhiteTailedDeer

MDPKGLLSLNVLLFFSLIFELSCRTGEGLTSSTKMILGQLGSSVLLPLASKEISRSMNKSIHILVTMAESPKDTVKKKIVSLDLRKGDSPRLEDGYEFHLENLSLRILKSKKEDEGWYFMSLEENVSVQQFSLQLKLYEQVSIPQIKVLNSTQEDGNCSLKLACMVEKGDHVTYNWSEEAGTPLLSPTNSSHLLYLTLGPQHANNVYICTVSNPISNSSQTFIPWSSCSSSPPESRQWGLYAGLFLGGIVGIIMILQVVILLLRRRGKTDNCQPTMEAKSLTIYAQVQKSGSVQKKPDPLPAQDPCTTIYVAATEPVPEPVQESGSFTVYASVTLPES*

>HogDeer

MDPKGRFSLNVLLFFSLIFELSCRTGEGLTSSTKTILGQLGSSVLLPLASKEISRSMNKSIHILVTMAESPKETVKKKIVSLDLRKGDSPRLEDGYEFHLENLSLRILKSKKEDEGWYFMSLEENVSVQQFSLQLKLYEQVSIPQIKVLNSTEEDGNCSLKLACMVEKGDHVTYNWSEEAGTPLLSPTNSSHLLYLTLGPQHANNVYICTVSNPISNSSQTFIPWSSCSSSSPESRQWGLYVGLFLGGIVGIIMILQVVILLLRRRGKTDNYQPTMEAKSLTIYAQVQKSGSVQKKPDPLPAQDPCTTIYVAATEPVPEPVQESGSFTVYASVTLPES*

>EasternRoeDeer

MDPKGLLSLNVLLFFSLIFELSCRTGEGLTSSTKTILGQLGSSVLLPLASKEISRSMNKSIHILVTMAESPKDTVKKKIVSLDLRKGDSPRLEDGYEFHLENLSLRILKSKKEDEGWYFMSLEENVSVQQFSLQLKLYEQVSIPQIKVLNSTQEDGNCSLKLACMVEKGDHVTYNWSEEAGTPLLSPANSSHLLYLTLGPQHANNVYICTVSNPISNSSQTFIPWSSCSSSPPESRQWGLYAGLFLGGIVGIIMILQVVILLLRRRGKTDNYQPTMEAKSLTIYAQVQKSGSVQKKPDPLPAQDPCTTIYVAATEPVPEPIQESGSFTVYASVTLPES*

>PereDavidsDeer

MDPKGRLSLNVLLFFSLIFELSCRTGEGLTSSTKTILGQLGSSVLLPLASKEISRSMNKSIHILVTMAESPKDTVKKKIVSLDLRKGDSPRLEDGYEFHLENLSLRILKSKKEDEGWYFMSLEENVSVQQFSLQLKLYEQVSIPQIKVLNSTQEDGNCSLKLACMVEKGDHVTYNWSEEAGTPLLSPTNSSHLLYLTLGPQHANNVYICTVSNPISNSSQTFIPWSSCSSSPPESRQWGLYAGLFLGGIVGIIMILQVVILLLRRRGKTDNYQPTMEAKSLTIYAQVQKSGSVQKKPDPLPAQDPCTTIYVAATEPVPEPVQESGSFTVYASVTLPES*

>MuleDeer

MDPKGLLSLNVLLFFSLIFELSCRTGEGLTSSTKMILGQLGSSVLLPLASKEISRSMNKSIHILVTMAESPKDTVKKKIVSLDLRKGDSPRLEDGYEFHLENLSLRILKSKKEDEGWYFMSLEENVSVQQFSLQLKLYEQVSTPQIKVLNSTQEDGNCSLKLACMVEKGDHVTYNWSEEAGTPLLSPTNSSHLLYLTLGPQHANNVYICTVSNPISNSSQTFIPWSSCSSSPPESRQWGLYAGLFLGGIVGIIMILQVVILLLRRRGKTDNCQPTMEAKSLTIYAQVQKSGSVQKKPDPLPAQDPCTTIYVAATEPVPEPVQESGSFTVYASVTLPES*

>ReevesMuntjac

MDPKGRLSLNVLLFFSLIFELSFRTGEGLTSSTKTILGQLGSSVLLPLASKEISRSMNKSIHILVTMAESPKDTVKKKIVSLDLRKGDSPRLEDGYEFHLENLSLRILKSKKEDEGWYFMSLEENVSVQQFSLQLKLYEQVSIPQIKVLNSTQEDGNCSLKLACMVEKGDHVTYNWSEEAGTPLLSPTNSSHLLYLTLGPQHANNVYVCTVTNPISNSSQTFIPWSSCSSSPPESRQWGLYAGLFLGGIVGIIMILQVVILLLRRRGKTDNHQPTMEAKSLTIYAQVQKSGSVQKKPDPLPAQDPCTTIYVAATEPVPEPVQESGSFTVYASVTLPES*

>WhiteLippedDeer

MDPKGRLSLNVLLFFSLIFELSCRTGEGLTSSTKTILGQLGSSVLLPLASKEISRSMNKSIHILVTMAESPKDTVKKKIVSLDLRKGDSPRLEDGYEFHLENLSLRILKSKKEDEGWYFMSLEENVSVQQFSLQLKLYEQVSIPQIKVLNSTQEDGNCSLKLACMVEKGDHVTYNWSEEAGTPLLSPTNSSHLLYLTLGPQHANNVYICTVSNPISNSSQTFIPWSSCSSSPPESRQWGLYAGLFLGGIVGIIMILQVVILLLRRRGKTDNYQPTMEAKSLTIYAQVQKSGSVQKKPDPLPAQDPCTTIYVAATEPVPEPVQESGSFTVYASVTLPES*

>EurasianElk

MDPKGLLSLNVLLFFSLIFELSCRTGEGLTSSTKTILGQLGSSVLLPLASKEISRSMNKSIHILVTMAESPKDTVKKKIVSLDLRKGDSPRLEDGYEFHLENLSLRILKSKQVSIPQIKVLNSTQEDGNCSLKLACMVEKGDHVTYNWSEEAGSPLLSPANSSHLLYLTLGPQHANNVYICTVSNPISNSSQTFIPWSSCSSSPPESRQWGLYAGLFLGGIVGIIMILQVVILLLRRRGKTDNYQPTMEAKSLTIYAQVQKSGSVQKKPDPLPAQDPCTTIYVAATEPVPEPVQESGSFTVYASVTLPES*

>IndianMuntjac

MDPKGCLSLNVLLFFSLIFELSFRTGEGLTSSTKTILGQLGSSVLLPLASKEISRSMNKSIHILVTMAESPKDTVKKKIVSLDLRKGDSPRLEDGYEFHLENLSLRILKSKKEDEGWYFMSLEENVSVQQFSLQLKLYEQVSIPQIKVLNSTQEDGNCSLKLACMVEKGDHVTYNWSEEAGTPLLSPTNSSHLLYLTLGPQHANNVYVCTVTNPISNSSQTFIPWSSCSSSPPESRQWGLYAGLFLGGIVGIIMILQVVILLLRRRGKTDNHQPTMEAKSLTIYAQVQKSGSVQKKPDPLPAQDPCTTIYVAATEPVPEPVQESGSFTVYASVTLPES*

>BighornSheep

MDPKGLLSSNVLLFFSLIIELSCRTGEGLTSSTKTIRGQLGSSVLLPLA?EEISRSMNKSIHILVTMAESPRDTVKKKIVSLDLRKGGSPRLEDGYEFHLENLSLRILKSRKEDEGWYFISLEENVSVQHFSLQLKLYEQVSTPQIKVLNSTQEDGNCSLMLA?MVEKGDHVTYNWSEEAGTPLLSPTNSSHLLYLTLGPQHANNVYICIASNPISNSSQTFI?W?R?SSRP?ESRQWGLYTGLF?GGIVGM?HDSPSGDTTVEKKR*NRQLPANNGSKKPYYLCPSPEIRFHSEET*PPASTGPLHHHLCRCHRACPRAHPGIRFLHSLCQCDASREL

>SableAntelope

MDPKGFLSSNVLLFFSLIIELSCRTGEGLTSSTKMVRGQLGSSVLLPLASEEISRSMNKSIHILVTMAESPKDTVKKKIVSLDLRKGGFPRLEDGYEFHLENLSLRILKSRKEDEGWYFISLEENVSVQHFSLQLKLYEQVSTPLIKVLNSTQEDGNCSLMLACMVEKGDHVTYNWSEEAGTPLLSPTNSSHLLYLTLGPQHANNVYICIASNPISNSSQTFIPWSRCSSRPPESRQWGLYAGLFLGGIVGIIMILQVVILLLRRRGKTDNYQPTMEAKSLTIYAQVQKSGSIQKKPDPLPAQDPCTTIYVAATEPVPEPIQESGSFTVYASVTLPES*

>Lechwe

MDPKGLLSSNVLLFFSLIIELSCRKGEGLTSSTKTILGQLGSSVLLPLASEEISRSMNKSIHILVTMAESPKDTVKKKIVSLDLRKGGSPRLEDGYEFHLENLSLRILKSRKEDEGWYFISLEENVSVQHFSLQLKLYEQVSTPQIKVLNSTQKDGNCSLMLACVVEKGDHVTYNWSEEAGTPLLSPTNSSHLLYLTLGPQHANNVYICIASNPISNSSQTFIPWSRCSSSPPESRQWGLYAGLFLGGIVGIIMILQVVILLLRRRGKTDNYQPTMEAKSLTIYAQVQKSGSIQKKPDPLAAQDPCTTIYVAATEPVPEPIQESGSFTVYASVTLPES*

>GrantsGazelle

MDPKGLLSNVLLFSSLIFELSCKTGEGLPSSTKTILGQLGSSVLLPLASEEISRSMNKSIHILVTMAESPRDTVKKKIVSLDLRKGGSPRLEDGYEFHLENLSLRILKSRKEDEGWYFISLEENVSVQHFSLQLKLYEQVSTPQIKVLNSTQEDGNCSLMLACMVEKGDHVTYNWIEEAGAPLLSPTNSSHLLYLTLGPQHANNVYICNVSNPISNSSQTFIPWSRCSSKSPEPKQWGLYVGLFLGSIVGIIMILQVVILLLRRRGKTDNCQPTMEAKSLTIYAQVQKSGSVQKKPDPLPAQDPCTTIYVAATEPVPEPIQESGSFTVYASVTLPES*

>RoyalAntelope

MDPKGLISSNAVLFFSLIFELSCRKGEGLTSSTKTILGQLGSSVLLPLASEEISRSMNKSIHILVTKAESPKDTVKKKIVSLDLQKGGSPRLEDGYEFHLENLSLRILKSRKEDEGWYFLSLEENVSVQHFSLQLKLYEQVSTPQIKVLNSTQEDGNCSLMLACMVEKGDHVTYNWSEEAGAPLLSPTNSSHLLYLTLGPQHANNVYICIVSNPISNSSQTFIPWSRCSSSPPESRQWGLYAGLFLGGIVGIIMILQVVILLLRRRGKTDNCQPTMEAKSLTIYAQVQKSGSIQKKPDSLPAQDPCTTIYVAATEPVPEPIQESGSFTVYASVTLPES*

>LesserKudu

MDPKGLLSSNVLLFFSLFVELSCRTGEGLTSSTKTILGQLGSSVLLPLASEEISRSMNKSIHILVTMAESPKDTVKKKIVSLDLRKGGSPRLEDGYEFHLENLSLRILKSRKEDEGWYFISLEENVSVQHFSLQLKLYEQVSTPQIKVLNSSQEDGNCSLMLACMVEKGDHVTYNWSEESGAPLLSPTNSSHLLYLTLGPQHANNVYICAVSNPISNSSQTFIPWPSCSSSPPESRQWGLYAGLFLGGIVGIIMILQVVILLLRRRGKTDNYQPATEAKSLTIYAQVQTSGSVQKKPDPLPAQDPCTTIYVAATEPVPEPIQESGSFTVYASVTPPES*

>Gayal

MDPKGLLSSNVLLLFSLIIELSCRTGEGLTSSTKTILGQLGSSVLLPLASEEISRSMNKSIHILVTMAESPKDTVKKKIVSLDLRKGDSPRLNDGYEFHLENLSLRILKSRKEDEGWYFISLEENVSVQHFSLQLKLYEQVSTPQIKVLNTTQEDGNCSLMLACMVEKGDHVTYNWSEEAGVPLLSPTNSSHLLYLTLGPQHANNVYICTVSNPISNSSQTFIPWPSCSSSPPESRQWGLYAGLFLGGIVGIIMILQVVILLLRRRGKTDNYQPTTEAKSLTIYAQVQTSGSVQKKPDPLPAQDPCTTIYVAATEPVPEPIQESGSFTVYASVTLPES*

>KirksDikDik

MDPKGLLSNVLLFSSLIFELSCRTGEGLPSSTKTILGQLGSSVLLPLASEEISRSMNKSIHILVTMAESPKDTVKKKIVS*ICGKAALHVWRTAMSFIWKT*A*GS*RAGRRMKAGTS*AWRKTFQSSTLACS*SSMSRSPLRKLRC*TPPRKMGTAVSCWPAWWRKGTT*LTTGVRKQAPPC*VPPTAPTSCISLLALSMPTTSTSAV*ATPSATALRPSSLGPGAAPGPQNQDNGDYMLGSS*GASLALS*FSKW*YYC*EEEVKQTIASQEWKQKALLSMPKSRNQVPFRRNRTPCQHRTPAPPFMSLPQSLSQSPSRNQVPSQSMPV*RFQRA

>MountainNyala

MDPKGLLSSNVLLFFSLFVELSCRTGEGLTSSTKTILGQLGSSVLLPLASEEISRSMNKSIHILVTMAESPKDTVKKKIVSLDLRKGGSPRLEDGYEFHLENLSLRILKSRKEDEGWYFISLEENVSVQHFSLQLKLYEQVSTPQIKVLNSSQEDGNCSLMLACMVEKGDHVTYNWSEEAGAPLLSPTNSSHLLYLTLGPQHANNVYICAVSNPISNSSQTFIPWPSCSSSPPVILQVVILLLRRRALLSMPKSRHQVLFRRNSTPCQHRTPVPPFMSLPQSLSQSPSRNQVPSRSMPV*RLQRA

>MountainGoat

MDPKGLLSSNVLLFFSLIIELSCRTGEGLTSSTKTIRGQLGSSVLLPLASEEISRSMNKSIHILVTMAESPRDTVKKKIVSLDLRKGGSPRLEDGYEFHLENLSLRILKSRKEDEGWYFISLEENVSVQHFSLQLKLYEQVSTPQIKVLNSTQEDGNCSLMLACVVEKGDHVTYNWSEEAGTPLLSPTNSSHLLYLTLGPQHANNVYICIASNPISNSSQTFIPWSSCSSRPPESRQWGLYAGLFLGGIVGIIMILQVVILLLRRRGKTDNYQPTMEAKSLTIYAQVQKSGSIQKRPDPLPAQDPCTTIYVAATEPVPEPIQESGSFTVYASVTLPES*

>YarkandDeer

MDPKGRLSLNVLLFFSLIFELSCRTGEGLTSSTKTILGQLGSSVLLPLASKEISRSMNKSIHILVTMAESPKDTVKKKIVSLDLRKGDSPRLEDGYEFHLENLSLRILKSKKEDEGWYFMSLEENVSVQQFSLQLKLYEQVSIPQIKVLNSTQEDGNCSLKLACMVEKGDHVTYNWSEEAGTPLLSPTNSSHLLYLTLGPQHANNVYICTVSNPISNSSQTFIPWSSCSSSPPESRQWGLYAGLFLGGIVGIIMILQVVILLLRRRGKTDNYQPTMEAKSLTIYAQVQKSGSVQKKPDPLPAQDPCTTIYVAATEPVPEPVQESGSFTVYASVTLPES*

>ChineseWaterDeer

MDPKGLLSLNVLLFFSLIFELSCRTGEGLTSSTKTILGQLGSSVLLPLASKEISRSMNKSIHILVTMAESPKDTVKKKIVSLDLRKGDSPRLEDGYEFHLENLSLRILKSKKEDEGWYFMSLEENVSVQQFSLQLKLYEQVSIPQIKVLNSTQEDGNCSLKLACMVEKGDHVTYNWSEEAGTPLLSPANSSHLLYLTLGPQHANNVYICTVSNPISNSSQTFIPWSSCSSSPPESRQWGLYAGLFLGGIVGIIMILQVVILLLRRRGKTDNYQPTMEAKSLTIYAQVQKSGSVQKKPDPLPAQDPCTTIYVAATEPVPEPVQESGSFTVYASVTLPES*

>BlackMuntjac

MDPKGHLSLNVLLFFSLIFELSFRTGEGLTSSTKTILGQLGSSVLLPLASKEISRSMNKSIHILVTMADHPNRVKKKIVSLDLRKVTLHVWRMAMSFIWKT*V*GS*RAKRRMKAGTLLSLEENVSVQQFSLQLKLYEQVSIPQIKVLNSTQEDGNCSLKLACMVEKGDHVTYNWSEEAGTPLLSPTNSSHLLYLTLGPQHANNVYVCTVTNPISNSSQTFIPWSSCSSSPPESRQWGLYAGLFLGGIVGIIMILQVVILLLRRRGKTDNHQPTMEAKSLTIYAQVQKSGESGSFTVYASVTLPES*

>SiberianMuskDeer

MDPKGLLSLNVLLFSSLILELSCRTGEGLTSSTKTILGQLGSSVLLPLASEEISRSMNKSIHILVTMAESPKDTVKKKIVSLDLRKGDSPRLEDGYEFHLENLSLRILKSRKEDEGWYFMSLEENVSVQHFSLQLKLYEQVSTPQIKVLNSTQKDGNCSVMLACTVEKGDHVTYNWSEEAGAPLLNPANGSHLLYLTLGPQHASNVYICTVSNPISNSSQTFIPWSSCSSSPPESRQWGLYAGLFLGGIVGIIMILQVVVLLLRRRGKTDNYQPTMEAKSLTIYAQVQKSGSVQKKPDPLPGQDPCTTIYVSATEPVPEPMQESGSFTVYASVTLPES*

>AlpineMuskDeer

MDPKGLLSLNVLLFSSLILELSCRTGEGLTSSTKTILGQLGSSVLLPLASEEISRSMNKSIHILVTMAESPKDTVKKKIVSLDLRKGDSPRLEDGYEFHLENLSLRILKSRKEDEGWYFMSLEENVSVQHFSLQLKLYEQVSTPQIKVLNSTQKDGNCSVMLACTVEKGDHVTYNWSEEAGAPLLNPANGSHLLYLTLGPQHASNVYICTVSNPISNSSQTFIPWSSCSSSPPESRQWGLYAGLFLGGIVGIIMILQVVVLLLRRRGKTDNYQPTMEAKSLTIYAQVQKSGSVQKKPDPLPAQDPCTTIYVSATEPVPGPMQESGSFTVYASVTLPES*

>WaterBuffalo

MDPKGLLSSNVLLLFSLIVELSCRTGEGLTSSTKTILGQLGSSVLLPLASEEISRSMNKSIHILVTMAESPKDTVKKKIVSLDLRKGGSPRLEDGYEFHLENLSLRILKSRKEDEGWYFISLEENVSVQHFSLQLKLYEQVSTPQIKVLNTTQEDGNCSLMLACMVEKGDHVTYNWSEEAGVPLLSPTNSSHLLYLTLGPQHANNIYICTVSNPISNSSQTFIPWPSCSSSPPESRQWGLYVGLFLGSIVGIIMILQVVILLLRRRGKTDNYQPTTEAKSLTIYAQVQTSGSVQKKPDPLAAQDPCTTIYVAATEPVPEPIQESGSFTVYASVTLPES*

>Gaur

MDPKGLLSSNVLLLFSLIIELSCRTGEGLTSSTKTILGQLGSSVLLPLASEEISRSMNKSIHILVTMAESPKDTVKKKIVSLDLRKGGSPPLKDGYEFHLENLSLRILKSRKEDEGWYFISLEENVSVQHFSLQLKLYEQVSTPQIKVLNTTQEDGNCSLMLACMVEKGDHVTYNWSEEAGVPLLSPTNSSHLLYLTLGPQHANNVYICTVSNPISNSSQTFIPWPSCSSSAPESRQWGLYAGLFLGGIVGIIMILQVVILLLRRRGKTDNYQPTTEAKSLTIYAQVQTSGSVQKKPDPLPAQDPCTTIYVAATEPVPEPIQESGSFTVYASVTLPES*

>CommonEland

MDPKGLLSSNVLLFFSLFVELSCRTGEGLTSSTKTILGQLGSSVLLPLASEEISRSMNKSIHILVTMAESPKDTVKKKIVSLDLRKGGSPRLEDGYEFHLENLSLRILKSRKEDEGWYFISLEENVSVQHFSLQLKLYEQVSTPQIKVLNSSQEDGNCSLMLACMVEKGDHVTYNWSEEAGTPLLSPTNSSHLLYLTLGPQHANNVYICAVSNPISNSSQTFIPWPSCSSSPPESRQWGLYAGLFLGGIVGIIMILQVVILLLRRRGKTDNYQPATEAKSLTIYAQVQTSGSVQKKPDPLPAQDPCTTIYVAATEPVPEPIQESGSFTVYASVTPPES*

>Bongo

MDPKGLLSSNVLLFFSLFVELSCRTGEGLTSSTKTILGQLGSSVLLPLASEEISRSMNKSIHILVTMAESPKDTVKKKIVSLDLRKGGSPRLEDGYEFHLENLSLRILKSRKEDEGWYFISLEENVSVQHFSLQLKLYDVTYNWSEEAGAPLLSPTNSSHLLYLTLGPQHANNVYICAVSNPISNSSQTFIPWPSCSSSPPVCWALLRGHRWHYHDSPSGDTTVEKKR*NRQLPASNGSKKPYYLCPSPDIRFCSEETRPPASTGPLHHHLCRCHRACPRAHPGIRFLHGLCQCDASREL

>MaxwellsDuiker

MDPKGLLSSNVLLFFSLIFELSCRTGEGLTSSTKTILGQLGSSVLLPLASEEISRSMNKSIHILVTMAESPKDTVKKKIVSLDLRKGGSPRLEDGYEFHLENLSLRILKSRKEDEGWYFISLEENVSVQHFSLQLKLYEQVSTPQIKVLNYTQKDGNCSLMLACVVEKGDHVTYNWSEEAGAPLLSPTNSSHLLYLTLGPQHANNVYICIVSNPISNSSQTVIPWSRCSSSPPESRQWGLYAGLFLGGIVGIIMILQVVILLLRRRGKTDNCQPTMEAKSLTIYAQVQKSGSDQKKPEPLPAQDPCTTIYVAATEPVPEPIQESGSFTVYASVTLPES*

>Gerenuk

MDPKGLLSNVLLFSSLIFELSCRTGEGLPSSTKTILGQLGSSVLLPLASEEISRSMNKSIHILVTMAESPRDTVKKKIVSLDLRKGGSPRLEDGYEFHLENLSLRILKSRKEDEGWYFISLEENVSVQHFSLQLKLYEQVSTPQIKVLNSTQEDGNCSLMLACMVEKGDHVTYNWSEEAGTPLLSPTNSSHLLYLTLGPQHANNVYICSVSNPISNSSQTFIPWSRCSSRSPESRQWGLYAGLFLGSIVGIIMILQVVILLLRRRGKTDNCQPTMEAKSLTIYAQVQKSGSVQKKPDPLPAQDPCTTIYVAATEPVPEPIQESGSFTVYASVTLPES*

>BlueWildebeest

MDPKGLLSSNVLLFFSLIIELSCRTGEGLTSSTKTIRGQLGSSVLLPLASEEISRSMNKSIHILVTMAESPKDTVKKKIVSLDLRKGGSPRLEDGYEFHLENLSLRILKSRKEDEGWYFISLEENVSVQHFSLQLKLYEQVSTPQIKVLNSTQEDGNCSLMLACMVEKGDHVTYNWSEEAGTPLLSPTNSSHLLYLTLGPQHANNVYICIASNPISNSSQTFIPWSRCSSRPPESRQWGLYAGLFLGGIVGIIMILQVVILLLRRRGKTDNYQPTMEAKSLTIYAQVQKSGSIQKKPDPLPAEDPCTTIYVAATEPVPEPIQESGSFTVYASVTLPES*

>ScimitarHornedOryx

MDPKGLLSSNVLLFFSLIIELSCRTGEGLTSSTKTVRGQLGSSVLLPLASEEISRSMNKSIHILVTMAESPKDTVKKKIVSLDLRKGGSPRLEDGYEFHLENLSLRILKSRKEDEGWYFISLEENVSVQHFSLQLKLYEQVSTPQIKVLNSTQEDGNCSLTLACMVEKGDHVTYNWSEEAGTPLLSPTNSSHLLYLTLGPQHANNVYICIASNPISNSSQTFIPWSRCSSRPPESRQWGLYAGLFLGGIVGIIMILQVVILLLRRRGKTDNYQPTMEAKSLTIYAQVQKSGSIQKKPDPLPAQDPCTTIYVAATEPVPEPIQESGSFTVYASVTLPES*

>AsiaticMouflon

MDPKGLLSSNVLLFFSLIIELSCRTGEGLTSSTKTIRGQLGSSVLLPLASEEISRSMNKSIHILVTMAESPRDTVKKKIVSLDLRKGGSPHLEDGYEFHLENLSLRILKSRKEDEGWYFISLEENVSVQHFSLQLKLYEQVSTPQIKVLNSTQEDGNCSLMLACMVEKGDHVTYNWSEEAGTPLLSPTNSSHLLYLTLGPQHANNVYICIASNPISNSSQTFIPWSRCSSRPPESRQWGLYTGLFLGGIVGIIMILQVVILLLRRRGKTDNCQPTMEAKSLTIYAQVQKSGSIQKRPDPLPAQDPCTTIYVAATEPVPEPIQESGSFTVYASVTLPES*

>Bharal

MDPKGLLSSNVLLFFSLIIELSCRTGEGLTSSTKTIRGQLGSSVLLPLASEEISRSMNKSIHILVTMAESPRDTVKKKIVSLDLRKGDSPHLEDGYEFHLENLSLRILKSRKEDEGWYFISLEENVSVQHFSLQLKLYEQVSTPQIKVLNSTQQDGNCSLMLACVVEKGDHVTYNWSEEAGAPLLSPTNSSHLLYLTLGPQHANNVYICIASNPISNSSQTFIPWSRCSSRPPESRQWGLYTGLFLGGIVGVIMILQVVILLLRRRGKTDNYQPTMEAKSLTIYAQVQKSGSIQKRPDPLPAQDPCTTIYVAATEPVPEPIQESGSFTVYASVRLPES*

>AfricanBuffalo

MDPKGLLSSNVLLLFSLIVELSCRTGEGLTSSTKTILGQLGSSVLLPLASEEISRSMNKSIHILVTMAESPKDTVKKKIVSLDLRKGGSPRLEDGYEFHLENLSLRILKSRKEDEGWYFISLEENVSVQHFSLQLKLYEQVSTPQIKVLNTTQEDGNCSLMLACMVEKGDHVTYNWSEEAGVPLLSPTNSSHLLYLTLGPQHANNVYICTVSNPISNSSQTFIPWPSCSSSPPESRQWGLYVGLFLGSIVGIIMILQVVILLLRRRGKTDNYQPTTEAKSLTIYAQVQTSGSVQKKPDPLPAQDPCTTIYVAATEPVPEPIQESGSFTVYASVTLPES*

>AmericanBison

MDPKGLLSSNVLLLFSLIIELSCRTGEGLTSSTKTILGQLGSSVLLPLASEEISRSMNKSIHILVTMAESPKDTVKKKIVSLDLRKGGSPPLKDGYEFHLENLSLRILKSRKEDEGWYFISLEENVSVQHFSLQLKLYEQVSTPQIKVLNTTQEDGNCSLMLACMVEKGDHVTYNWSEEAGVPLLSPTNSSHLLYLTLGPQHANNVYICTVSNPISNSSQTFIPWPSCSSSPPESRQWGLYAGLFLGGIVGIIMILQVVILLLRRRGKTDNYQPTTEAKSLTIYAQVQTSGSVQKKPDPLPAQDPCTTIYVAATEPVPEPIQESGSFTVYASVTLPES*

>GreaterKudu

MDPKGLLSSNVLLFFSLFVELSCRTGEGLTSSTKTILGQLGSSVLLPLASEEISRSMNKSIHILVTMAESPKDTVKKKIVSLDLRKGGSPRLEDGYEFHLENLSLRILKSRKEDEGWYFISLEENVSVQHFSLQLKLYEQVSTPQIKVLNSSQEDGNCSLMLACMVEKGDHVTYNWSEEAGAPLLSPTNSSHLLYLTLGPQHANNVYICTVSNPISNSSQTFIPWPSCSSSPPESRQWGLYAGLFLGGIVGIIMILQVVILLLRRRGKTDNYQPATEAKSLTIYAQVQTSGSVQKKPDPLPAQDPCTTIYVAATEPVPEPIQESGSFTVYASVMPPES*

>Suni

MDPKGLLSSNVLLFFSLIIELSCRTGEGLTSSTKTILGQLGSSVLLPLASEEISRSMNKSIHILVTMAESPKDTVKKKIVSLDLRKGGSPRGGWL*VSSGKPEPEDPEEQEGG*RLVLYKPGGKCFSPAL*PAAEAL*AGLHSAN*GVELHPGRWELQSHAGLRGGERGPRDLQLE*GGRHPPAESHQ*LPPPISHSWPSACQQRLHLHREQPHQQQLSDLHPLVQVQLQSPRIKTMGTICWALLRGHRWHYHDSPSGDTTVEKKR*NRQLSANNGSKEPYYLCPSPEIRFGSEET*PPASTGPLHHHLCRCHRACPRAHPGIRFLHSLCQCDASREL

>HarveysDuiker

MDPKGLLSSNVLLFFSLIFELSCRTGEGLTSSTKMILGQLGSSVLLPLASEEISRSMNKSIHILVTMAESPKDTVKKKIVSLDLRKGGSPRLEDGYEFHLENLSLRILKSRKEDEGWYFISLEENVSVQHFSLQLKLYEQVSTPQIKVLNSTREDGNCSLMLACVVEKGDHVTYNWSEEAGTPLLSPTNSSHLLYLTLGPQHANNVYICIVSNPISNSSQTVIPWSRCSSSPPESRQWGLYAGLFLGGIVGIIMILQVVILLLRRRGKTDNCQPTMEAKSLTIYAQVQKSGSDQKKPEPLPAQDPCTTIYVAATEPVPEPTQESGSFTVYASVTLPES*

>Oribi

MDPKGLLSNVLLFSSLIFELSCRTGEGLPSSTKTILGQLGSSVLLPLASEEISRSMNKSIHILVTMAESPRDTVKKKIVSLDLRKGGSPRLEDGYEFHLENLSLRILNSRKEDEGWYFISLEENVSVQHFSLQLKLYEQVSTPQIKVLNSTREDGNCSLMLACVVEKGDHVTYNWSEEAGAPLLSPTNSSHLLYLTLGPQHANSVYICSVSNPISSSSQTLIPWSRCSSRSPESRQWGLYAGLFLGSIVGIIMILQVVILLLRRRGKTDNCQPTMEAKSLTIYAQVQKSGSVQKKPDPLPAQDPCTTIYVAATEPVPEPIQESGSFTVYASVTLPES*

>Springbok

MDPKGLLSNVLLFSSLIFELSCRTGEGLPSSTKTILGQLGSSVLLPLASEEISRSMNKSIHILVTMAESPGDTVKKKIVSLDLRKGGSPRGGRL*VSSGKPEPEDPEEQEGG*RLVLHKPGGKCFSPAL*PAAEAL*AGLHSAN*GVELHPGRWELQSHAGLHGGERGPRDLQLE*GSRRPPAEPHQ*LPPLVSHSWPSACRQRLHLQCEQPHQQQLSDLHPLVQVQLRVPRIKTMGTICWALLREHRWHYHDSPSGDSTVEKKR*NRQLPANNGSKKPYYLCPSPEIRFLSEET*PPASTGPLHHHLCRCHRACPRAHPGIRFLHSLCQCDASREL

>Herola

MDPKGLLSSNVLLFFSLIIELSCRTGEGLTSSTKTIRGQLGSSVLLPLASEEISRSMNKSIHILVTMAESPKDTVKKKIVSLDLRKGGSPRLEDGYEFHLENLSLRILKSRKEDEGWYFISLEENVSVQHFSLQLKLYEQVSTPQIKVLNSTQEDGNCSLMLACMVEKGDHVTYNWSEEAGTPLLSPTNSSHLLYLTLGPQHANNVYICIASNPISNSSQTFIPWSRCSSRPPESRQWGLYAGLFLGGIVGIIMILQVVILLLRRRGKTDNYQPTMEAKSLTIYAQVQKSGSVQKKPDPLPAQDPCTTIYVAATEPVPEPIQESGSFTVYASVTLPES*

>Gemsbok

MDPKGLLSSNVLLFFSLIIELSCRTGEGLTSSTKTVRGQLGSSVLLPLASEEISRSMNKSIHILVTMAESPKDTVKKKIVSLDLRKGGSPRLEDGYEFHLENLSLRILKSRKEDEGWYFISLEENVSVQHFSLQLKLYEQVSTPQIKVLNSTQEDGNCSLTLACMVEKGDHVTYNWSDEAGTPLLSPTNSSHLLYLTLGPQHANNVYICIASNPISNSSQTFIPWSRCSSRPPESRQWGLYAGLFLGGIVGIIMILQVVILLLRRRGKTDNYQPTMEAKSLTIYAQVQKSGSIQKKPDPLPAQDPCTTIYVAATEPVPETIQESGSFTVYASVTLPES*

>EuropeanMouflon

MDPKGLLSSNVLLFFSLIIELSCRTGEGLTSSTKTIRGQLGSSVLLPLASEEISRSMNKSIHILVTMAESPRDTVKKKIVSLDLRKGGSPHLEDGYEFHLENLSLRILKSRKEDEGWYFISLEENVSVQHFSLQLKLYEQVSTPQIKVLNSTQEDGNCSLMLACMVEKGDHVTYNWSEEAGTPLLSPTNSSHLLYLTLGPQHANNVYICIASNPISNSSQTFIPWSRCSSRPPESRQWGLYTGLFLGGIVGIIMILQVVILLLRRRGKTDNCQPTMEAKSLTIYAQVQKSGSIQKRPDPLPAQDPCTTIYVAATEPVPEPIQESGSFTVYASVTLPES*

>NilgiriTahr

MDPKGLLSSNVLLFFSLIIELSCRTGEGLTSSTKTIRGQLGSSVLLPLASEEISRSMNKSIHILVTMAESPRDTVKKKIVSLDLRKGGSPHLEDGYEFHLENLSLRILKSRKEDEGWYFISLEENVSVQHFSLQLKLYEQVSTPQIKVLNSTQEDGNCSLMLACMVEKGDHVTYNWSEEAGAPLLSPTNSSHLLYLTLGPQHANNVYICIASNPISNSSQTFIPWSRCSSRPPESRQWGLYTGLFLGGIVGIIMILQVVILLLRRRGKTDNYQPTMEAKSLTIYAQVQKSGSIQKRPDPLPAQDPCTTIYVAATEPVPEPIQESGSFTVYASVTLPES*

>ZebuCattle

MDPKGLLSSNVLLLFSLIIELSCRTGEGLTSSTKTILGQLGSSVLLPLASEEISRSMNKSIHILVTMAESPKDTVKKKIVSLDLRKGDSPRLNDGYEFHLENLSLRILKSRKEDEGWYFISLEENVSVQHFSLQLKLYEQVSTPQIKVLNTTQEDGNCSLMLACMVEKGDHVTYNWSEEAGVPLLSPTNSSHLLYLTLGPQHANNVYICTVSNPISNSSQTFIPWPSCSSSAPESRQWGLYAGLFLGGIVGNIMVLQVVILLLRRRGKTDNYQPTTEAKSLTIYAQVQTSGSVQKKPDPLPAQDPCTTIYVAATEPVPEPIQESGSFTVYASVTLPES*

>DomesticYak

MDPKGLLSSNVLLLFSLIIELSCRTGEGLTSSTKTILGQLGSSVLLPLASEEISRSMNKSIHILVTMAESPKDTVKKKIVSLDLRKGGSPPLKDGYEFHLENLSLRILKSRKEDEGWYFISLEENVSVQHFSLQLKLYEQVSTPQIKVLNTTQEDGNCSLMLACMVEKGDHVTYNWSEEAGVPLLSPTNSSHLLYLTLGPQHANNVYICTVSNPISNSSQTFIPWPSCSSSPPESRQWGLYAGLFLGGIVGIIMILQVVILLLRRRGKTDNYQPTTEAKSLTIYAQVQTSGSVQKKPDPLPAQDPCTTIYVAATEPVPEPIQESGSFTVYASVTLPES*

>Bushbuck

MDPKGLLSSNVLLFFSLFVELSCRTGEGLTSSTKTILGQLGSSVLLPLASEEISRSMNKSIHILVTMAESPKDTVKKKIVSLDLRKGGSPRLEDGYEFHLENLSLRILKSRKEDEGWYFISLEENVSVQHFSLQLKLYEQVSTPQIKVLNSSQEDGNCSLMLACMVEKGDHVTYNWSEEAGAPLLSPTNSSHLLYLTLGPQHANNVYICAVSNPISNSSQTFIPWPSCSSSPPESRQWGLYAGLFLGGIVGIIMILQVVILLLRRRGKTDNYQPATEAKSLTIYAQVQTSGSVQKKLDPLPAQDPCTTIYVAATEPVPEPIQESGSFTVYASVTPPES*

>Impala

MDPKGLLSLNVLLFFSLIIELSCRTGEGLTSSTKTILGQLGSSVLLPLASEEISRSMNKSIHILVTMAESPKDTVKKKIVSLDLRKGGSPRLEHGYEFHLENLSLRILKSRKEDEGWYFISLEENVSVQHFSLQLKLYEQVSTPEIKVLNSTQEDGNCSLMLACVVEKGDHVTYNWSEEAGTPLLSPTNSSHLLYLTLGPQQANNVYICIVSNPISNSSQIFIPWSSCSSSPPESRQWGLYAGLFLGGIVGIIMILQVVILLLRRRGKTDNCQPTMEAKSLTIYAQVQKSGSIQKKPEPLPAQDPCTTIYVAATEPVPEPIQESASFTVYASVTLPES*

>CommonDuiker

MDPKGLLSSNVLLFFSLIFELSCRTGEGLTSSTKTILGQLGSSVLLPLASEEISRSMNKSIHILVTMAESPKDTVKKKIVSLDLRKGGSPRLEDGYEFHLENLSLRILKSRKEDEGWYFISLEENVSVQHFSLQLKLYEQVSTPQIKVLNSTREDGNCSLMLACVVEKGDHVTYNWSEEAGTPLLSPTNSSHLLYLTLGPQHANNVYICIVSNPISNSSQTVIPWSRCSSRPPESRQWGLYAGLFLGGIVGIIMILQVVILLLRRRGKTDNCQPTMEAKSLTIYAQVQKSGSDQKKPEPLPAQDPCTTIYVAATEPVPEPIQESGSFTVYASVTLPES*

>Saiga

MDPKGLLLNVLLFFSLIYELSCRTGEGLPSSTKTILGQLGSSVLLPLASEEISRSMNKSIHILVTKAESPRDTVKKKIVSLDLRKGGSPRLEDGYEFHLENLSLRILKSRKEDEGWYFVSLEENVSVQHFSLQLKLYEQVSTPQIKVLNSTQEDGNCSLMLACVVEKGDHVTYNWSEEAGSPLLSPTNSSHLLYLTLGPQHANNVYICSVSNPISNSSQTFIPWSRCSSRSPESRQWGLYAGLFLGSIVGIIMILQVVILLLRRRGKTDNCQPTVEAKSLTIYAQVQKSGSIQKKPDPLPAQDPCTTIYVAATEPVPEPIQESGSFTVYASVTLPES*

>BohorReedbuck

MDPKGLLSSNVLLFFSLIIELSCRKGEGLTSSTKTILGQLGSSVLLPLASEEISRSMNKSIHILVTMAESPKDTVKKKIVSLDLRKGGSPRLEDGYEFHLENLSLRILKSRKEDEGWYFISLEENVSVQHFSLQLKLYEQVSTPQIKVLNSTQKDGNCSLMLACVVEKGDHVTYNWSEEAGTPLLSPTNSSHLLYLTLGPQHANNVYICIASNPISNSSQTFFPWSRCSSSPPESRQWGLYAGLFLGGIVGIIMILQVVILLLRRRGKTDNYQPTMEAKSLTIYAQVQKSGSIQKKPDPLAAQDPCTTIYVAATEPVPEPIQESGSFTVYASVTLPES*

>Topi

MDPKGLLSSNVLLFFSLIIELSCRTGEGLTSSTKTIRGQLGSSVLLPLASEEISRSMNKSIHILVTMAESPKDTVKKKIVSLDLRKGGSPRLEDGYEFHLENLSLRILKSRKEDEGWYFISLEENVSVQHFSLQLKLYEQVSTPQIKVLNSTQEDGNCSLMLACMVEKGDHVTYNWSEEAGTPLLSPTNSSHLLYLTLGPQHANNVYICIASNPISNSSQTFIPWSRCSSRPPESRQWGLYAGLFLGGIVGIIMILQVVILLLRRRGKTDNYQPTMEAKSLTIYAQVQKSGSVQKKPDPLPAQDPCTTIYVAATEPVPEPIQESGSFTVYASVTLPES*

>TibetanAntelope

MDPKGLLSLNVLLFFSLIIELSCRTGESLTSSAKTILGQLGSSVLLPLASEEISRNMNKSIHILVTTAESPKDTVKKKIVSLDLRKGGSPRLEDGYEFHLENLSLRILKSKKEDEGWYFISLEENVSVQHFSLQLKLYEQVSTPQIKVLNSTQEDGNCSLMLACMVEKGDHVTYNWSEEAGAPLLSPTNSSHLLYLTLGPQHANNVYICIASNPISNSSQTFIPWSRCSSRPSDSRQWGLYAGLFLGGIIGIIMILQVVILLLRRRGKTDNYQPTMEAKSLTIYAQVQKSGSIQKKPDPLPAQDPCTTIYVAATEPVPEPIQESGSFTVYASVTLPES*

>SiberianIbex

MDPKGLLSSNVLLFFSLIIELSCRTGEGLTSSTKTIRGQLGSSVLLPLASEEISRSMNKSIHILVTMAESPRDTVKKKIVSLDLRKGGSPRLEDGYEFHLENLSLRILKSRKEDEGWYFISLEENVSVQHFSLQLKLYEQVSTPQIKVLNSTQEDGNCSLMLACVVEKGDHVTYNWSEEAGAPLLSPTNSSHLLYLTLGPQHANNVYICIASNPISNSSQTFIPWSRCSSRPPESRQWGLYTGLFLGGIVGVIMILQVVILLLRRRGKTDNYQPTMEAKSLTIYAQVQKSGSIQKRPDPLPAQDPCTTIYVAATEPVPEPIQESGSFTVYASVTVPES*

>WildYak

MDPKGLLSSNVLLLFSLIIELSCRTGEGLTSSTKTILGQLGSSVLLPLASEEISRSMNKSIHILVTMAESPKDTVKKKIVSLDLRKGGSPPLKDGYEFHLENLSLRILKSRKEDEGWYFISLEENVSVQHFSLQLKLYEQVSTPQIKVLNTTQEDGNCSLMLACMVEKGDHVTYNWSEEAGVPLLSPTNSSHLLYLTLGPQHANNVYICTVSNPISNSSQTFIPWPSCSSSPPESRQWGLYAGLFLGGIVGIIMILQVVILLLRRRGKTDNYQPTTEAKSLTIYAQVQTSGSVQKKPDPLPAQDPCTTIYVAATEPVPEPIQESGSFTVYASVTLPES*

>Sitatunga

MDPKGLLSSNVLLFFSLFVELSCRTGEGLTSSTKTILGQLGSSVLLPLASEEISRSMNKSIHILVTMAESPKDTVKKKIVSLDLRKGGSPRLEDGYEFHLENLSLRILKSRKEDEGWYFISLEENVSVQHFSLQLKLYEQVSTPQIKVLNSSQEDGNCSLMLACMVEKGDHVTYNWSEEAGAPLLSPTNSSHLLYLTLGPQHANNVYICAVSNPISNSSQTFIPWPSCSSSPPESRQWGLYAGLFLGGIVGIIMILQVVILLLRRRGKTDNYQPATEAKSLTIYAQVQTSGSVQKKPDPLPAQDPCTTIYVAATEPVPEPIQESGSFTVYASVTPPES*

>Klipspringer

VRA*PVPQRRFSGSWEAACCCPWQLRR*VGA*IRASTSLSQWQNHPQTLSRRK*CP*ICGKVALHVWRMAMSFIWKT*A*GS*RAGRRMKAGTL*AWRKMFQSSTLACS*SSMSRSPLRKLRC*TPPRKMGTAVSCWPAWWKKGTT*LTTGVRKQVPPC*VPPIAPTSCISLLALSMPTTSTSAL*ATPSATALRPSSLGPGAAPVPQNQDNGDYMLGSS*GASLALS*FSKW*YYY*EEEVKQTIASQQWKQKALLSMPKSRNQVPFRRNLTPCQHRTPAPPFTSLPQSLSQSPSRNQVPSQSMPV*RFQRA

>PrzewalskisGazelle

VRACPVPQRRFSGSWEAACCCPWHLRR*AGA*IRASTSSSQWQNHP*TPSRRKYCP*ICGKVALHIWRMSMSFIWKT*A*GS*RAGRRMKAGTS*PWRKMFQSSTLACS*SSMIRSPLRKLRC*TPPRKMGTAVSCWPAWWRKGTM*LTTGVRKQAPPC*VPPTAPTSCISLLALSMPTTSTSAV*ATPSATALRHSSLGPGAAPGPQNQGNGDYMLGSS*GASLALS*FSKW*YYC*EEEVKQTIASQQRKQKALLSMPKSRNQVPFRRNLAPCQHRTPAPPFMSLPQSPSQSPSRNQVPSQSMPV*RFQRA

>ThomsonsGazelle

MDPKGLLSNVLLFSSLIFELSCRTGEGLPSSTKTILGQLGSSVLLPLASEEISRSMNKSIHILVTMAESPRDTVKKKIVSLDLRKGDSPRLEDGYEFHLENLSLRILKSRKEDEGWYFISLEENVSVQHFSLQLKLYEQVSTPQINVLNSTQEDGNCSLMLACVVEKGDHVTYNWSEEAGAPLLSPTNSSHLLYLTLGPQHANNVYICSVSNPISNSSQTFIPWSRCSSRSSDSKQWGLYAGLFLGSIVGIILILQVVILLLRRRGKTDNCQPTMEEKSLTIYAQVQKSGSVQKKPDPLPAQDPCTTIYVAATEPVPEPTQESGSFTVYASVTLPES*

>DefassaWaterbuck

MDPKGLLSSNVLLFFSLIIELSCRKGEGLTSSTKTILGQLGSSVLLPLASEEISRSMNKSIHILVTMAESPKDTVKKKIVSLDLRKGGSPLEDGYEFHLENLSLRILKSRKEDEGWYFISLEENVSVQHFSLQLKLYEQVSTPQIKVLNSTQKDGNCSLMLACVVEKGDHVTYNWSEEAGTPLLSPTNSSHLLYLTLGPQHANNVYICIASNPISNSSQTFIPWSRCSSSPPESRQWGLYAGLFLGGIVGIIMILQVVILLLRRRGKTDNYQPTMEAKSLTIYAQVQKSGSIQKKPDPLAAQDPCTTIYVAATEPVPEPIQESGSFTVYASVTLPES*

>RoanAntelope

MDPKGLLSSNVLLFFSLIIELSCRTGEGLTSSTKMVRGQLGSSVLLPLASEEISRSMNKSIHILVTMAESPKDTVKKKIVSLDLRKGGSPRLEDGYEFHLENLSLRILKSRKEDEGWYFISLEENVSVQHFSLQLKLYEQVSTPLIKVLNSTQEDGNCSLMLACMVEKGDHVTYNWSEEAGTPLLSPTNSSHLLYLTLGPQHANNVYICIASNPISNSSQTFIPWSRCSSRPPESRQWGLYAGLFLGGIVGIIMILQVVILLLRRRGKTDNYQPTMEAKSLTIYAQVQKSGSIQKKPDPLPAQDPCTTIYVAATEPVPEPIQESGSFTVYASVTLPES*

>JavaMouseDeer

MDPRGLPSLNVLLFFFLAIELTCRTGEGLMSSTKMVLGRLGSSVLLPLAPEEISRSMNKSIHILVTMAESPKDTVKKKIVSLDLRKGDSPHLEKGYEFHLENLSLRILKSRKEDEGWYFTSLEENISVQHFSLQLKLYEQVSTPKIKVLNSSQENGNCSLILACMVEKGDHVTYNWSEEAGDPLLSPANDSHLLYLTLGPQHTDNIYMCTVSNPISNSSQTFSPWSRCSSSPPESRQWGLFVGLFLGSIVGVIMILQVVVLLLRRRGKTDNYQPTVEAKSLTIYAQVQKSGSIQKKPDPLPAQDHCTTIYVAATEPVPEPAQESSSFTVYASVTLPES*

>Pronghorn

MDPKGLLSLNVLLLFELSCRPGEGLTSSTKTILGQLGSSVLLPLASEEISGSMNKSIHILVTMAESPKDTVKKKIVSLDLRKGDSPRLEDGYEFHLENLSLRILKSRKEDAGWYFMSLEENVSVQHFSLQLKLYEQVSTPQIKVLNVTQEDGNCSLMLACTVEKGDHVTYNWSEEAGTPLLSPTNSSHLVHLTLGPRHVNNVYICTVSNPISNSSQTFIPWSSCSSTSPESRQWGLYAGLFLGGIVGIIMILQVVILLLRRRGKTDNYQPAMEAKSLTIYAQVQKSGSIQKKPDPLPAQDPCTTIYVAATEPVPEPIQESGSFTVYASVTLPES*

>Okapi

MDPKGLLSLNVLLLFSLIFELNCRTGEGFTSSTKTILGQLGSSVLLPLASEEISRRMNKSIHILVTMAESPKDTVKKKIVSLDLRKGDSPRLEDGYEFHLENLSLSILKSKREDAGWYFMSLEENVSVQHFSLQLKLYEQVSTPQIKVLNSTQEGGNCSLMLACMVEKGDHVTYNWNEEAGTPLLSPTNSSPHLLSLILGPQLANSVYICTVSNPISNSSQTFIPWSSCSSSPPESRQWGLYAGLFLGGIVGIIMILQVVILLLRRRGKTDNYQPTMEAKSLTIYAQVQKSGSVQKKPDPLPAQDPCTTIYVAATEPVPEPIQESGSFTVYASVTLPES*

>RedDeer

MDPKGRLSLNVLLFFSLIFELSCRTGEGLTSSTKTILGQLGSSVLLPLASKEISRSMNKSIHILVTMAESPKETVKKKIVSLDLRKGDSPRLEDGYEFHLENLSLRILKSKKEDEGWYFMSLEENVSVQQFSLQLKLYEQVSIPQIKVLNSTQEDGNCSLKLACMVEKGDHVTYNWSEEAGTPLLSPTNSSHLLYLTLGPQHANNVYICTVSNPISNSSQTFIPWSSCSSSPPESRQWGLYAGLFLGGIVGIIMILQVVILLLRRRGKTDNYQPTMEAKSLTIYAQVQKSGSVQKKPDPLPAQDPCTTIYVAATEPVPEPVQESGSFTVYASVTLPES*

>ChineseForestMuskDeer

MDPKGLLSLNVLLFSSLILELSCRTGEGLTSSTKTILGQLGSSVLLPLASEEISRSMNKSIHILVTMAESPKDTVKKKIVSLDLRKGDSPRLEDGYEFHLENLSLRILKSRKEDEGWYFMSLEENVSVQHFSLQLKLYEQVSTPQIKVLNSTQKDGNCSVMLACTVEKGDHVTYNWSEEAGAPLLNPANGSHLLYLTLGPQHASNVYICTVSNPISNSSQTFIPWSSCSSSPPESRQWGLYAGLFLGGIVGIIMILQVVVLLLRRRGKTDNYQPTMEAKSLTIYAQVQKSGSVQKKPDPLPAQDPCTTIYVSATEPVPGPVQESGSFTVYASVTLPES*

>BarbarySheep

MDPKGLLSSNVLLFFSLIIELSCRTGEGLTSSTKTIRGQLGSSVLLPLASEEISRSMNKSIHILVTMAESPRDTVKKKIVSLDLRKGGSPRLEDGYEFHLENLSLRILKSRKEDEGWYFISLEENVSVQHFSLQLKLYEQVSTPQIKVLNSTQEDGNCSLMLACMVEKGDHVTYNWSEEAGSPLLSPTNSSHLLYLTLGPQHANNVYICIASNPISNSSQTFIPWSRCSSRPPESRQWGLYTGLFLGGIVGVIMILQVVILLLRRRGKTDNYQPTVEAKSLTIYAQVQKSGSIQKRPDPLPAQDPCTTIYVAATEPVPEPIQESGSFTVYASVTLPES*

>SnowSheep

MDPKGLLSSNVLLFFSLIIELSCRTGEGLTSSTKTIRGQLGSSVLLPLASEEISRSMNKSIHILVTMAESPRDTVKKKIVSLDLRKGGSPRLEDGYEFHLENLSLRILKSRKEDEGWYFISLEENVSVQHFSLQLKLYEQVSTPQIKVLNSTQEDGNCSLMLACMVEKGDHVTYNWSEEAGTPLLSPTNSSHLLYLTLGPQHANNVYICIASNPISNSSQTFIPWSRCSSRPPESRQWGLYTGLFLGGIVGIIMILQVVILLLRRRGKTDNYQPTMEAKSLTIYAQVQKSGSIQKRPDPLPAQDPCTTIYVAATEPVPEPIQESGSFTVYASVTLPES*

>WildGoat

MDPKGLLSSNVLLFFSLIIELSCRTGEGLTSSTKTIRGQLGSSVLLPLASEEISRSMNKSIHILVTMAESPRDTVKKKIVSLDLRKGDSPRLEDGYEFHLENLSLRILKSRKEDEGWYFISLEENVSVQHFSLQLKLYEQVSTPQIKVLNSTQEDGNCSLMLACVVEKGDHVTYNWSEEAGAPLLSPTNSSHLLYLTLGPQHANNVYICIASNPISNSSQTFIPWSRCSSRPPESRQWGLYTGLFLGGIVGVIMILQVVILLLRRRGKTDNYQPTMEAKSLTIYAQVQKSGSIQKRPDPLPAQDPCTTIYVAATEPVPEPIQESGSFTVYASVTVPES*

>Cattle

MDPKGLLSSNVLLLFSLIIELSCRTGEGLTSSTKTILGQLGSSVLLPLASEEISRSMNKSIHILVTMAESPKDTVKKKIVSLDLRKGDSPRLNDGYEFHLENLSLRILKSRKEDEGWYFISLEENVSVQHFSLQLKLYEQVSTPQIKVLNTTQEDGNCSLMLACMVEKGDHVTYNWSEEAGVPLLSPTNSSHLLYLTLGPQHANNVYICTVSNPISNSSQTFIPWPSCSSSAPESRQWGLYAGLFLGGIVGIIMILQVVILLLRRRGKTDNYQPTTEAKSLTIYAQVQTSGSVQKKPDPLPAQDPCTTIYVAATEPVPEPIQESGSFTVYASVTLPES*

>Sheep

MDPKGLLSSNVLLFFSLIIELSCRTGEGLTSSTKTIRGQLGSSVLLPLASEEISRSMNKSIHILVTMAESPRDTVKKKIVSLDLRKGGSPHLEDGYEFHLENLSLRILKSRKEDEGWYFISLEENVSVQHFSLQLKLYEQVSTPQIKVLNSTQEDGNCSLMLACMVEKGDHVTYNWSEEAGTPLLSPTNSSHLLYLTLGPQHANNVYICIASNPISNSSQTFIPWSRCSSRPPESRQWGLYTGLFLGGIVGIIMILQVVILLLRRRGKTDNCQPTMEAKSLTIYAQVQKSGSIQKRPDPLPAQDPCTTIYVAATEPVPEPIQESGSFTVYASVTLPES*
